# Benchmarking monolayer MoS_2_ and WS_2_ field-effect transistors

**DOI:** 10.1038/s41467-020-20732-w

**Published:** 2021-01-29

**Authors:** Amritanand Sebastian, Rahul Pendurthi, Tanushree H. Choudhury, Joan M. Redwing, Saptarshi Das

**Affiliations:** 1 Department of Engineering Science and Mechanics, Penn State University, University Park, PA 16802 USA; 2 2D Crystal Consortium-Materials Innovation Platform (2DCC-MIP), Penn State University, University Park, PA 16802 USA; 3 Department of Materials Science and Engineering, Penn State University, University Park, PA 16802 USA; 4 Materials Research Institute, Penn State University, University Park, PA 16802 USA

**Keywords:** Electronic devices, Two-dimensional materials

## Abstract

Here we benchmark device-to-device variation in field-effect transistors (FETs) based on monolayer MoS_2_ and WS_2_ films grown using metal-organic chemical vapor deposition process. Our study involves 230 MoS_2_ FETs and 160 WS_2_ FETs with channel lengths ranging from 5 μm down to 100 nm. We use statistical measures to evaluate key FET performance indicators for benchmarking these two-dimensional (2D) transition metal dichalcogenide (TMD) monolayers against existing literature as well as ultra-thin body Si FETs. Our results show consistent performance of 2D FETs across 1 × 1 cm^2^ chips owing to high quality and uniform growth of these TMDs followed by clean transfer onto device substrates. We are able to demonstrate record high carrier mobility of 33 cm^2^ V^−1^ s^−1^ in WS_2_ FETs, which is a 1.5X improvement compared to the best reported in the literature. Our experimental demonstrations confirm the technological viability of 2D FETs in future integrated circuits.

## Abstract (web summary)

Here, the authors perform a benchmark study of field-effect transistors (FETs) based on 2D transition metal dichalcogenides, i.e., 230 MoS_2_ and 160 WS_2_ FETs, and track device-to-device variations to gauge the technological viability in future integrated circuits.

## Introduction

Two-dimensional (2D) semiconducting materials beyond graphene^1,2^ are receiving increasing attention owing to their ultra-thin body nature that can mitigate detrimental short-channel effects in aggressively scaled devices through improved electrostatics, enabling them to replace or complement the aging Si technology^3–5^. Molybdenum disulfide (MoS_2_) and tungsten disulfide (WS_2_), belonging to the family of transition metal dichalcogenides (TMDs), have been studied extensively in this context. In fact, high performance MoS_2_ field-effect transistors (FETs) with a contact pitch of 70 nm and 42 nm have already been experimentally demonstrated^6,7^. Circuit level implementations of 2D FETs such as inverters, logic operators, ring oscillators, and radio-frequency devices have also been achieved^8–12^. Recently, a microprocessor based on MoS_2_ FETs was reported^13^. Additionally, 2D FETs have found applications in various emerging technologies such as sensors for internet of things, neuromorphic computing, biomimetic devices, valleytronics, straintronics, and optoelectronic devices^14–21^. While initial demonstrations of prototype devices relied on exfoliated flakes, the 2D community has rapidly transitioned towards the growth of large-area films to address manufacturing needs for any commercial applications. In this context, chemical vapor deposition (CVD)^22,23^ and metal-organic CVD (MOCVD)^7,24^ are the most promising techniques, enabling growth of high quality 2D materials with different thermal budgets on various substrates. In fact, there are several reports demonstrating high-performance FETs based on CVD and MOCVD grown monolayer MoS_2_ and WS_2_. However, most of these studies are based on one or only a few devices.

To assess the potential of 2D materials for future very large scale integrated (VLSI) circuits, it is important to study the variation in key device parameters that determine the ON-state and OFF-state performance across a large number of devices. Unfortunately, there are only a few studies that report device-to-device variation in 2D FETs^7,25,26^. Smithe et al. measured multiple parameters across 200 MoS_2_ FETs and demonstrated low threshold voltage variation and low contact resistance on the order of 1 kΩ−μm^25^. Similarly. Xu et al. analyzed 380 top-gated MoS_2_ FETs and reported variation in threshold voltage and electron mobility^26^. However, both works concentrate on longer channel devices where the effects of contact resistance are not pronounced. In a separate study, Smithe et al.^22^ measured scaled MoS_2_ FETs based on synthetic monolayers; however, they did not provide any statistics. Smets et al.^7^ demonstrated the most significant study on scaling of CVD grown monolayer MoS_2_, wherein multiple devices with channel lengths ranging from 5 μm down to 29 nm were measured. However, their study was focused on the OFF-state performance. Finally, all of the aforementioned studies are based on MoS_2_ FETs, and none exist for WS_2_ FETs.

This work focuses on a comprehensive study of variation in key parameters related to both OFF-state and ON-state performance, such as threshold voltage, subthreshold slope, ratio of maximum to minimum current, field-effect carrier mobility, contact resistance, drive-current, and carrier saturation velocity, for different channel lengths ranging from 5 μm down to 100 nm using 230 MoS_2_ FETs and 160 WS_2_ FETs. In addition, we offer extensive benchmarking of our devices with respect to the above-mentioned demonstrations as well as ultra-thin body (UTB) silicon (Si) on insulator (SOI) FETs with similar gate lengths to assess the technological viability and maturity of 2D FETs. Using statistical measures such as mean, median, standard deviation, and minimum/maximum values, we show low device-to-device variation. We are also able to demonstrate record high carrier mobility of 33 cm^2^ V^−1^ s^−1^ in WS_2_ FETs, which is a 1.5X improvement compared to the best reported in the literature. We attribute our accomplishments to the epitaxial growth of highly crystalline 2D monolayers on sapphire substrate *via* MOCVD technique at 1000 °C using chalcogen and sulfur precursors that minimize carbon contamination in the film, as well as to the clean transfer of the film from the growth substrate to the device fabrication substrate.

## Results

### Synthesis and characterization of monolayer MoS_2_ and WS_2_

**Fig. 1:**
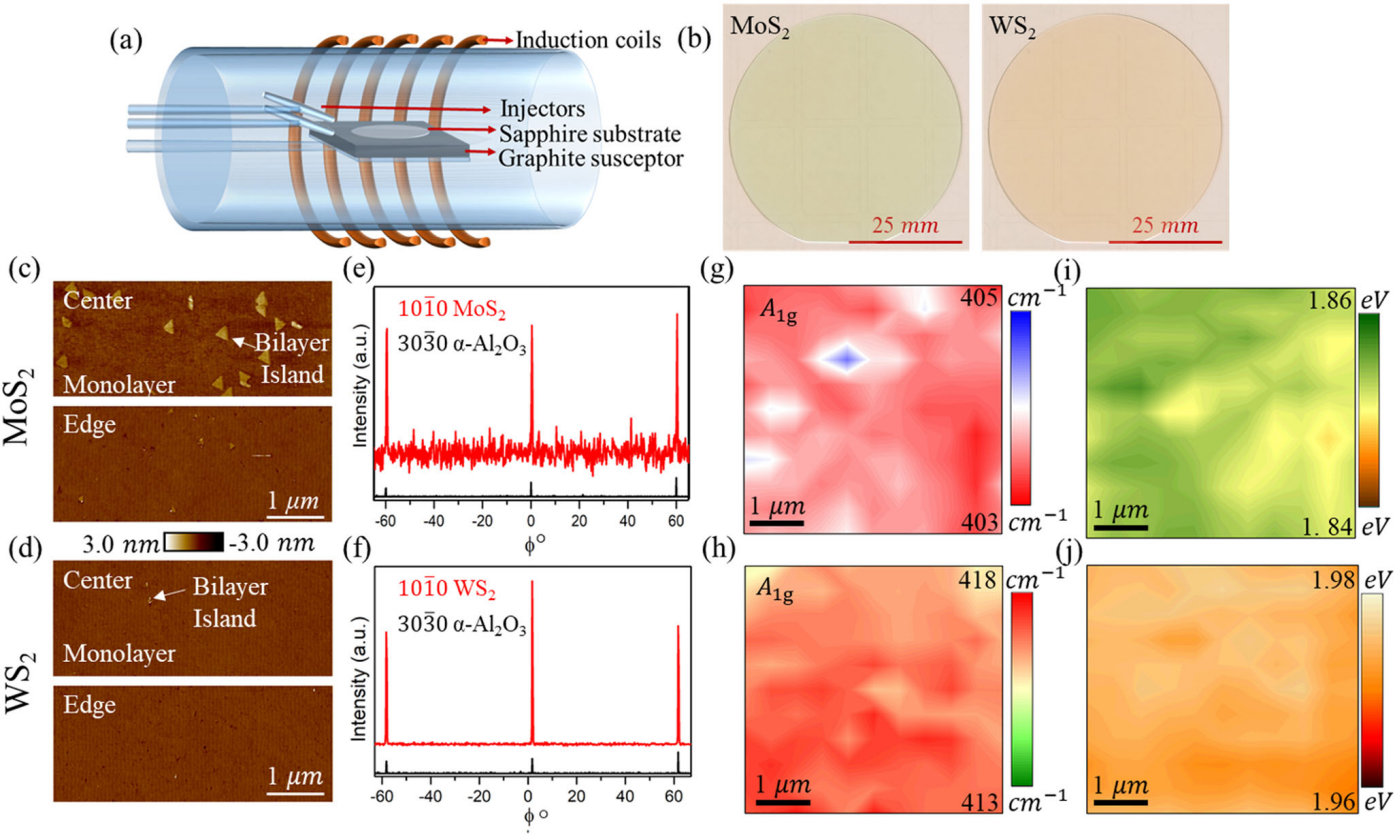
Monolayer film growth and characterization. **a** Schematic of the MOCVD system with a cold-wall horizontal reactor. **b** 2″ sapphire wafer with MOCVD grown MoS_2_ and WS_2_. AFM images of **c** MoS_2_ and **d** WS_2_ at the center and edge of the respective wafers. Towards the center of the MoS_2_ film, few bilayers are seen. In-plane XRD ϕ-scan of **e** MoS_2_ and **f** WS_2_ on sapphire (α-Al_2_O_3_), showing the epitaxial relationship between the monolayers and the sapphire substrate. Raman map of the *A*_1g_ peak position for **g** MoS_2_ and **h** WS_2_ films transferred from the growth substrate onto the device fabrication substrate with 50 nm ALD Al_2_O_3_. Low variation in the peak position is observed for both MoS_2_ and WS_2_ with an average of ≈403.5 cm^−1^ and ≈417 cm^−1^, respectively. PL peak map of **i** MoS_2_ with an average of ≈1.85 eV and **j** WS_2_ with an average of ≈1.97 eV, confirm monolayer films. PL is a characteristic of monolayer film owing to indirect to direct bandgap transition.

MoS_2_ and WS_2_ were deposited by MOCVD on epi-ready 2″ diameter c-plane sapphire wafers. Figure 1 summarizes the growth, structural, and optical characterization of the MOCVD grown MoS_2_ and WS_2_. Figure 1a shows the schematic of the MOCVD system, comprising of a cold-wall horizontal reactor with an inductively heated graphite susceptor equipped with wafer rotation as previously described^27^. Molybdenum hexacarbonyl (Mo(CO)_6_) and tungsten hexacarbonyl (W(CO)_6_) were used as metal precursors, while hydrogen sulfide (H_2_S) was the chalcogen source with H_2_ as the carrier gas. MoS_2_ was deposited in a single step process at 1000 °C, where coalesced monolayer growth across the 2″ wafer was achieved in 18 min. WS_2_ was deposited using a multi-step process with nucleation at 850 °C and lateral growth at 1000 °C, resulting in coalesced monolayer growth across the 2″ wafer in 10 min^28^. In both cases, after growth the substrate was cooled in H_2_S to 300 °C to inhibit decomposition of the MoS_2_ and WS_2_ films. Figure 1b shows uniformly grown MoS_2_ and WS_2_ films over 2″ sapphire wafers. Further growth details can be found in the “Methods” section. The morphology of the monolayer films at the center and edge of the 2″ wafer is shown in Fig. 1c, d for MoS_2_ and WS_2_, respectively, using atomic force microscopy (AFM). Height profiles obtained from scratch testing confirm monolayer film formation (see Supplementary Fig. 1a, b). The monolayers are fully coalesced, with undulations arising from steps on the sapphire surface. The overall bilayer density is low but a higher density of bilayers is present at the center of the MoS_2_ film compared to the WS_2_ film. The in-plane X-ray diffraction (XRD) patterns in Fig. 1e, f highlight the epitaxial relation between the sulfide monolayers and the underlying sapphire substrates. The full-width at half maxima of the ϕ-scan peaks are 0.3° and 0.17° for MoS_2_ and WS_2_, respectively, indicating a low rotational misorientation of domains within the monolayers. The films were transferred to Al_2_O_3_/Pt/TiN/p^++^-Si substrates for device fabrication, as discussed later. The transferred film quality was assessed using Raman maps as shown in Fig. 1g, h, and photoluminescence (PL) maps as shown in Fig. 1i, j, for MoS_2_ and WS_2_, respectively. Raman maps show less than 5% variation in the representative *A*_1g_ peak position. The uniform PL peak positions observed at 1.84 eV for MoS_2_ and 1.97 eV for WS_2_ correspond to their monolayer response. Representative Raman and PL spectra are included in the Supplementary Fig. 1c–f.

### Monolayer MoS_2_ and WS_2_ device fabrication and characterization

**Fig. 2:**
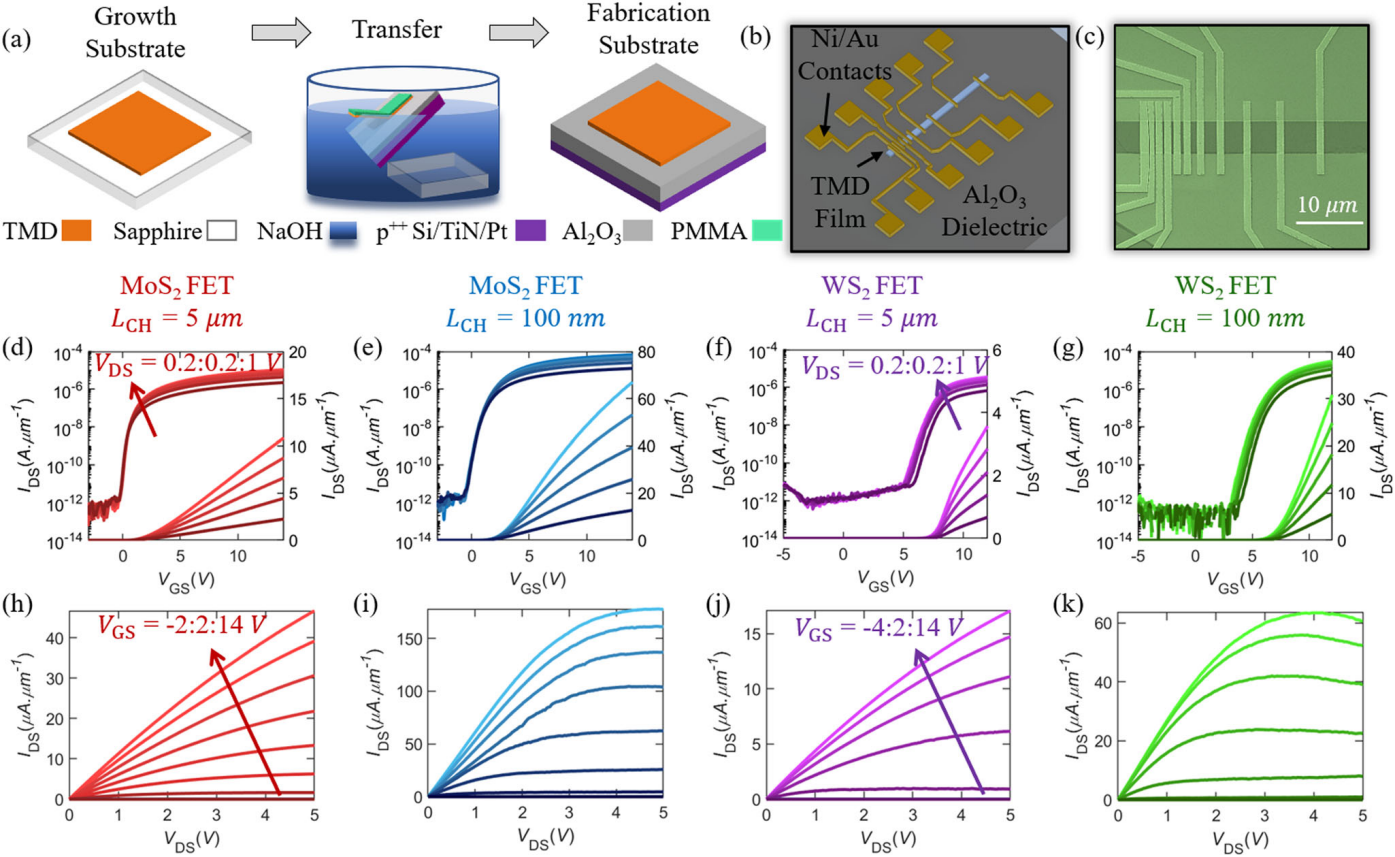
Device fabrication and electrical measurements. **a** Schematic representation of PMMA-assisted wet transfer of monolayer TMD films from sapphire (growth substrate) to an Al_2_O_3_ substrate. **b** Schematic representation and **c** SEM of a TLM structure defined using e-beam lithography. TLM structures with channel length (*L*_CH_) from 100 nm to 5 μm are fabricated and the channel width is defined to be 5 μm. Transfer characteristics, i.e., drain current (*I*_DS_) versus gate voltage (*V*_GS_), for different drain voltages (*V*_DS_) in logarithmic and linear scale for **d** longest-channel (*L*_CH_ = 5 μm) and **e** shortest-channel (*L*_CH_ = 100 nm) MoS_2_ field-effect transistors (FETs), and **f** longest-channel (*L*_CH_ = 5 μm) and **g** shortest-channel (*L*_CH_ = 100 nm) WS_2_ FETs. Corresponding output characteristics, i.e., *I*_DS_ versus *V*_DS_, for different *V*_GS_ for **h** longest-channel and **i** shortest-channel MoS_2_ FETs, and **j** longest-channel and **k** shortest-channel WS_2_ FETs.

To investigate the electrical properties of the MOCVD grown TMD films, back-gated FETs were fabricated on Al_2_O_3_/Pt/TiN/p^++^-Si substrates. 50 nm Al_2_O_3_ gate dielectric was deposited using atomic layer deposition (ALD). The choice of a thin, high-k gate dielectric with an effective oxide thickness (EOT) of 22 nm, compared to conventionally used 300 nm SiO_2_, was to allow for better gate electrostatics. The Pt/TiN/ p^++^-Si stack acts as the gate electrode (see “Methods” section for more details on gate dielectric fabrication) for each substrate. The TMD films were transferred from sapphire (growth substrates) onto the Al_2_O_3_ substrates *via* the poly(methyl methacrylate) (PMMA)-assisted wet-transfer process^29^, as shown in Fig. 2a (see “Methods” section for more details on transfer of monolayer films). Following transfer, electron beam (e-beam) lithography and dry etching using SF_6_ plasma were used to isolate the channel area of each device. Next, transmission line measurement (TLM) structures were defined using another set of e-beam exposures. Finally, e-beam evaporation was performed to sequentially deposit 40 nm Ni and 30 nm Au to serve as the contacts for the FETs (see “Methods” section for more details on device fabrication). The TLM structures were designed to have channel lengths (*L*_CH_) of 100 nm, 200 nm, 300 nm, 400 nm, 500 nm, 1 μm, 2 μm, 3 μm, 4 μm, and 5 μm, while the channel width (*W*) was kept constant at 5 μm. Figure 2b, c, respectively, show the schematic and scanning electron microscope (SEM) image of the fabricated TLM structures. Figure 2d–g show the transfer characteristics, i.e., drain current (*I*_DS_) versus gate voltage (*V*_GS_), for different drain voltages (*V*_DS_) in linear and logarithmic scales for representative longest-channel length (*L*_CH_ = 5 μm) and shortest-channel length (*L*_CH_ = 100 nm) FETs, for both MoS_2_ and WS_2_. Strong n-type conduction is observed due to Fermi-level pinning of the contact metal close to the conduction band of both MoS_2_ and WS_2_^30^. Figure 2h–k show the corresponding output characteristics, i.e., *I*_DS_ versus *V*_DS_, for different *V*_GS_. Measurement protocols are described in the “Methods” section.

### Device-to-device variation in monolayer MoS_2_ and WS_2_ FETs

**Fig. 3:**
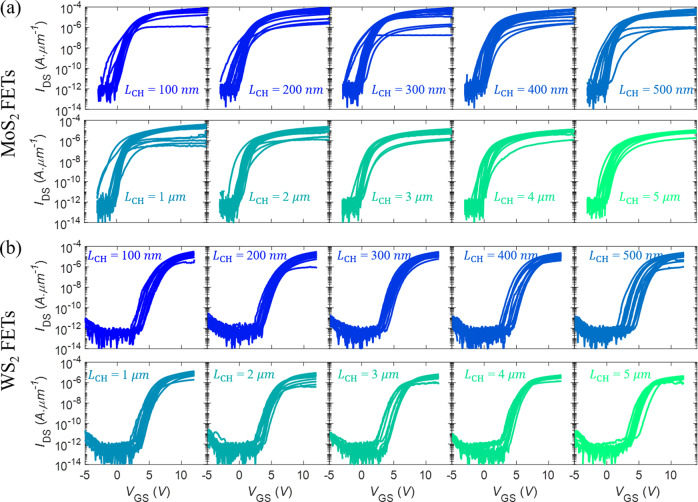
Statistics of scaled devices. A total of 230 MoS_2_ FETs and 160 WS_2_ FETs were fabricated using 23 and 16 TLM structures with channel lengths ranging from *L*_CH_ = 100 nm to *L*_CH_ = 5 μm for **a** MoS_2_ and **b** WS_2_, respectively, to analyze the device-to-device variation and impact of scaling on the device performance. The corresponding transfer characteristics, i.e., *I*_DS_ versus *V*_GS_, for *V*_DS_ of 1 V are shown in the logarithmic scale.

To understand the variation in the FET performance across the entire 1 × 1 cm^2^ substrates, as well as to study of the impact of channel length scaling on FET performance, 230 MoS_2_ FETs (23 TLM structures) and 160 WS_2_ FETs (16 TLM structures) were measured. Figure 3a, b display the transfer characteristics of all measured MoS_2_ and WS_2_ FETs, respectively, for different *L*_CH_, which were used to extract key device parameters. For each parameter, the mean, median, standard deviation, minimum, and maximum values are reported. Finally, median values are used for benchmarking since they reflect the central tendency, even in the presence of outliers in the data, and offer higher accuracy in case of skewed distributions. Devices with the best number for a given parameter are termed as “champion” devices.

#### Threshold voltage

**Fig. 4:**
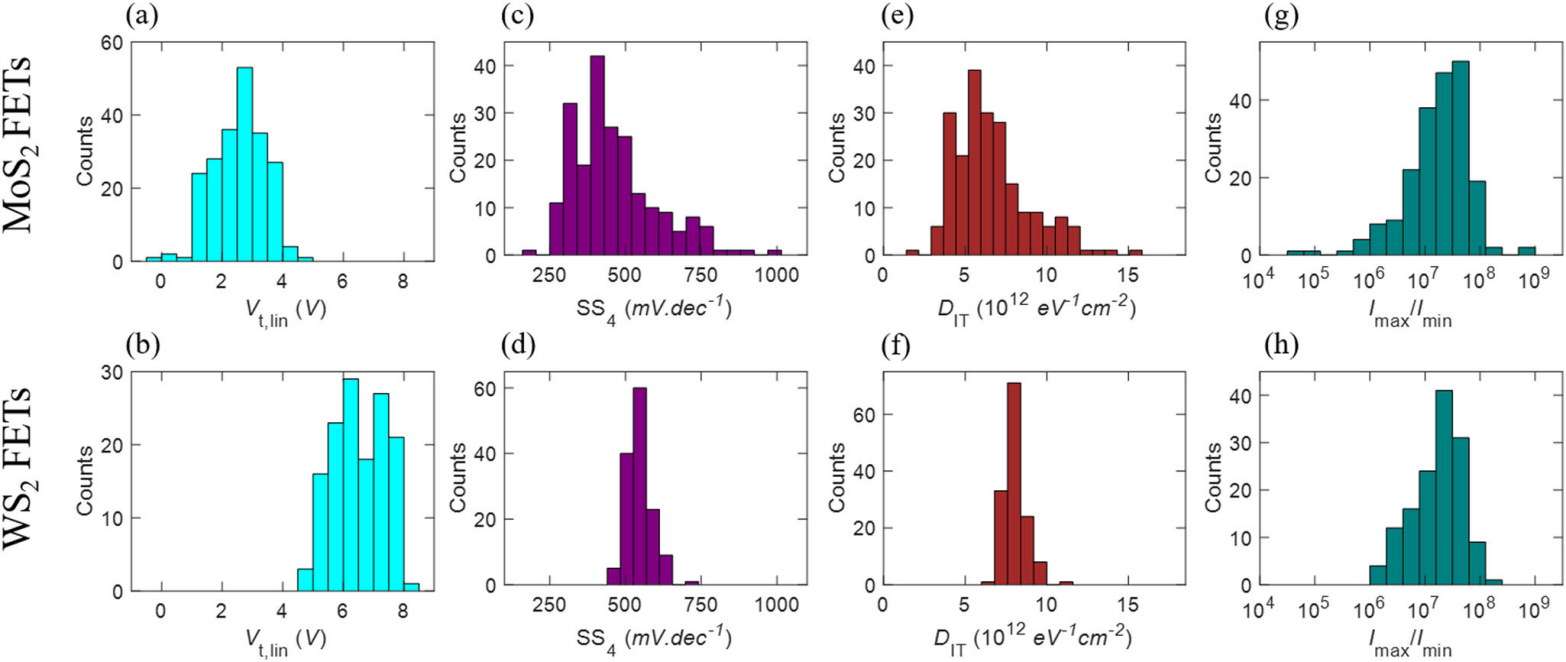
Variation in OFF-state performance. Histograms showing the variation in threshold voltage extracted using linear extrapolation (*V*_t,lin_) for **a** MoS_2_ and **b** WS_2_ FETs. The median values for these extracted threshold voltages were found to be more positive for WS_2_ FETs compared to MoS_2_ FETs due to higher intrinsic n-type doping of MoS_2_. Histograms of SS extracted across 4 orders of magnitude change in the drain current (SS_4_) for **c** MoS_2_ and **d** WS_2_ FETs. The deviation of SS from the ideal 60 mV.dec^−1^ corresponds to the presence of interface traps. Histograms of interface trap density (*D*_IT_) for **e** MoS_2_ and **f** WS_2_ FETs calculated from SS_4_. Histograms of maximum to minimum current ratio (*I*_max_/*I*_min_) for **g** MoS_2_ and **h** WS_2_ FETs.

Threshold voltage is extracted using three different methods: linear extrapolation ($V_{{\mathrm{t}}_{{\mathrm{lin}}}}$), Y-function ($V_{{\mathrm{t}}_Y}$), and constant-current method ($V_{{\mathrm{t}}_{{\mathrm{cc}}}}$). Supplementary Fig. 2a–c describes the extraction of $V_{{\mathrm{t}}_{{\mathrm{lin}}}}$, $V_{{\mathrm{t}}_Y}$, and $V_{{\mathrm{t}}_{{\mathrm{cc}}}}$, Supplementary Fig. 2d, e show their corresponding median values as a function of *L*_CH_, and Supplementary Fig. 2f, g show their distributions across all devices for MoS_2_ and WS_2_, respectively. Supplementary Note 1 and Supplementary Table 1 summarize the device-to-device variations. It was found that the threshold voltage is independent of the channel length for both MoS_2_ and WS_2_ FETs. Figure 4a, b show the distributions of $V_{{\mathrm{t}}_{{\mathrm{lin}}}}$ for all measured MoS_2_ and WS_2_ FETs, respectively. Median $V_{{\mathrm{t}}_{{\mathrm{lin}}}}$ of 2.9 V with a standard deviation of $\sigma _{V_{\mathrm{t}}}$ = 0.8 V is obtained for MoS_2_, and median $V_{{\mathrm{t}}_{{\mathrm{lin}}}}$ of 6.4 V with a $\sigma _{V_{\mathrm{t}}}$ = 0.8 V is obtained for WS_2_. Threshold voltage was found to be more positive for WS_2_ FETs compared to MoS_2_ FETs, which can be attributed to higher intrinsic n-type doping of MoS_2_ either due to the specific nature of the impurity present in the MOCVD grown MoS_2_ film or due to surface charge transfer induced doping due to the underlying ALD grown Al_2_O_3_. This charge transfer is accredited to the higher conduction band offset between MoS_2_ and Al_2_O_3_ compared to WS_2_ and Al_2_O_3_^31^.

Variation in threshold voltage is widely used for benchmarking emerging devices based on novel materials^25^. Note that median $V_{{\mathrm{t}}_{{\mathrm{lin}}}}$ depends on the work function of the gate metal and unintentional/intrinsic doping of the 2D material and that both $V_{{\mathrm{t}}_{{\mathrm{lin}}}}$ and $\sigma _{V_{\mathrm{t}}}$ depend on the thickness of the gate oxide. Hence for a fair comparison we use $S\sigma _{V_{\mathrm{t}}}$, which is defined as the projected threshold voltage variation at a scaled effective oxide thickness (SEOT) obtained using Eq. (1). We use SEOT = 0.9 nm for comparison with other literature results.

1 $$S\sigma _{V_{\mathrm{t}}} = \sigma _{V_{\mathrm{t}}}\frac{{{\mathrm{SEOT}}}}{{{\mathrm{EOT}}}}$$

This equation assumes linear scaling of variation in threshold voltage with respect to the EOT. However, for ultra-scaled devices, deviation from the linear scaling can be expected due to increased effect of metal-gate granularity^32^. For our MoS_2_ and WS_2_ FETs, we project $S\sigma _{V_{\mathrm{t}}}$ = 33 mV, which is similar to the value projected for CVD grown monolayer MoS_2_ FETs reported by Smithe et al.^25^. We also employed this method to other reports on top-gated and wafer-scale monolayer MoS_2_ FETs and extracted $S\sigma _{V_{\mathrm{t}}}$ = 45 mV for^26^ and $S\sigma _{V_{\mathrm{t}}}$ = 11 mV for^12^, respectively. Recently, Smets et al.^7^ have demonstrated $\sigma _{V_{\mathrm{t}}}$ = 44 mV for an EOT of 1.9 nm that would correspond to $S\sigma _{V_{\mathrm{t}}}$ = 20 mV for monolayer MoS_2_ FETs with channel lengths scaled down to 30 nm. These results are compared with the state-of-the-art UTB SOI and Si FinFET (Table 1). Channel dimensions are included in Table 1 since $\sigma _{V_{\mathrm{t}}}$ has been found to be inversely proportional to the channel area in ultra-scaled devices which is shown using Pelgrom plots^32,33^. However, we did not observe such a trend due to relatively large channel areas in our MoS_2_ and WS_2_ FETs. It is encouraging that monolayer 2D FETs show $S\sigma _{V_{\mathrm{t}}}$ comparable to the state-of-the art Si FETs in spite of an order of magnitude smaller body thickness. Note that UTB Si FETs are expected to encounter challenges associated with the precise thickness control, random dopant fluctuations, and detrimental quantum confinement effects beyond 5 nm body thickness^34,35^, which are unlikely for 2D monolayers. At the same time further improvement in threshold voltage variation can be achieved for 2D FETs through optimization of the monolayer growth and improvement in the fabrication process flow (see Supplementary Note 2 for further discussion). Hence, 2D materials offer an alternative for the realization of UTB MOSFETs. The exhibition of low device-to-device variation in this work, which can be attributed to uniform and contaminant-free MOCVD growth of monolayer TMDs and clean device fabrication process can accelerate the incorporation of 2D FETs in future VLSI technologies.

**Table 1:** Benchmarking device-to-device variation in threshold voltage.

	${\mathbf{\sigma}} _{V_{\mathrm{t}}}({\mathrm{V}})$	Gate dielectric	$S{\mathbf{\sigma}} _{V_{\mathrm{t}}}({\mathrm{V}})$ at SEOT = 0.9 nm	Channel dimensions (µm)
^25^—MoS_2_	1.05	30 nm SiO_2_	33 × 10^−3^	*W* = 11.6, *L*_CH_ = 4–8.6
^26^—MoS_2_ 1 continuous layer	0.25	30 nm HfO_2_	45 × 10^−3^	*W* = –, *L*_CH_ = 30
^26^—MoS_2_ 1 layer + ML	0.1	30 nm HfO_2_	19 × 10^−3^	*W* = –, *L*_CH_ = 30
^12^—MoS_2_	0.17	30 nm Al_2_O_3_	11 × 10^−3^	*W* = 30, *L*_CH_ = 4
^7^—MoS_2_	44 × 10^−3^	4 nm HfO_2_	20 × 10^−3^	*W* = 1, *L*_CH_ = 0.1
Our work-MoS_2_, WS_2_	0.8	50 nm Al_2_O_3_	33 × 10^−3^	*W* = 5, *L*_CH_ = 0.1, 0.2, 0.3, 0.4, 0.5, 1, 2, 3, 4, 5
^33^—UTB SOI	24.5 × 10^−3^	EOT = 1.65 nm	13 × 10^−3^	*W* = 0.060, *L*_CH_ = 0.025
^32^—FinFET	10 × 10^−3^	EOT = 0.8 nm	11 × 10^−3^	*W* = 0.0075, *L*_CH_ = 0.034

#### Subthreshold slope

Subthreshold slope (SS) is extracted over 1 (SS_1_), 2 (SS_2_), 3 (SS_3_), and 4 (SS_4_) orders of magnitude change in *I*_DS_ for MoS_2_ and WS_2_ FETs, respectively. Supplementary Fig. 3a, b show the median values for SS_1_, SS_2_, SS_3_, and SS_4_ as a function of *L*_CH_, and Supplementary Fig. 3c, d show the distributions for SS_1_ and SS_4_ for all MoS_2_ and WS_2_ devices, respectively. Supplementary Table 2 summarizes the device-to-device variation in SS. For a FET with ohmic contacts, it is expected that SS_1_ = SS_2_ = SS_3_ = SS_4_. However, for a Schottky barrier (SB) FET, the SS may increase when extracted for higher orders of magnitude change in *I*_DS_. A greater increase can be attributed to higher SB height at the metal/semiconductor interface, which not only limits the ON-current but also impacts the OFF-state performance. In the existing 2D FET literature there is a tendency to report SS value without mentioning the orders of magnitude change in *I*_DS_ over which it is evaluated. This leads to considerable discrepancy and unfair comparisons. In fact, most SS values are reported for only one or two orders of magnitude of the drain current, whereas circuit operations require at least four orders of magnitude ON/OFF ratio to be technologically relevant.

We found that the median SS values are independent of *L*_CH_ for both MoS_2_ and WS_2_ FETs (Supplementary Fig. 3a, b). Fig. 4c, d show the distributions of SS_4_ for all measured MoS_2_ and WS_2_ FETs, respectively. A median SS_4_ of 431.9 mV.dec^−1^ with a standard deviation of *σ*_SS_ = 138.1 mV.dec^−1^ is obtained for MoS_2_, and a median SS_4_ of 541.4 mV.dec^−1^ with a *σ*_SS_ = 41.8 mV.dec^−1^ is obtained for WS_2_. The median SS_4_ values show slight increase from the corresponding median SS_1_ values of 327.1 mV.dec^−1^ and 438.2 mV.dec^−1^ for MoS_2_ and WS_2_, respectively (Supplementary Table 2). However, no significant difference is found in the standard deviation values for SS_1_ and SS_4_. Note that the “champion” MoS_2_ FET demonstrates SS_1_ = 93.3 mV.dec^−1^ and SS_4_ = 166 mV.dec^−1^, and the “champion” WS_2_ FET demonstrates SS_1_ = 295.6 mV.dec^−1^ and SS_4_ = 452.8 mV.dec^−1^. The deviation of SS from its ideal value of 60 mV.dec^−1^even for “champion” devices can be explained using Eq. (2).

2 $${\mathrm{SS}} = \frac{{mk_{\mathrm{B}}T}}{q}{\mathrm{ln}}\left( {10} \right);m = \left( {1 + \frac{{C_{\mathrm{S}}}}{{C_{{\mathrm{OX}}}}} + \frac{{C_{{\mathrm{IT}}}}}{{C_{{\mathrm{OX}}}}}} \right);C_{{\mathrm{IT}}} = qD_{{\mathrm{IT}}}$$

Here, *k*_B_ is the Boltzmann constant, *T* is the temperature, *q* is the electronic charge, *m* is the body factor, *C*_S_ is the semiconductor capacitance, *C*_IT_ is the interface trap capacitance, *C*_OX_ is the oxide capacitance, and *D*_IT_ is the interface trap density. For fully depleted UTB FETs such as monolayer MoS_2_ and WS_2_ FETs, *C*_S_ = 0. In case of a clean oxide-semiconductor interface, $C_{{\mathrm{IT}}} \ll C_{{\mathrm{OX}}}$, ensuring that *m* = 1 and SS = 60 mV.dec^−1^. Clearly, in our MoS_2_ and WS_2_ FETs, *m* > 1 indicates the presence of interface traps at the 2D/dielectric interface (finite value of *C*_IT_).

#### Interface traps

To evaluate the quality of the interface, we have extracted *D*_IT_ using Eq. (2) and the corresponding distributions are shown in Fig. 4e, f for MoS_2_ and WS_2_ FETs, respectively. Median *D*_IT_ of 6.2 × 10^12^ eV^−1^ cm^−2^ and 8 × 10^12^ eV^−1^ cm^−2^ were obtained for MoS_2_ and WS_2_, respectively. The device-to-device variation in *D*_IT_ is shown in Supplementary Table 2*.* For fully depleted UTB Si MOSFETs with 35 nm thick Si and 110 nm gate length, SS = 80 mV.dec^−1^ for an EOT = 4 nm, which corresponds to a *D*_IT_ = 1.5 × 10^12^ eV^−1^ cm^−2^^36^. Note that, while the *D*_IT_ values for our monolayer 2D FETs are comparable with state-of-the-art Si FETs, thicker EOT = 22 nm results in smaller *C*_OX_ and hence higher median values for the *SS* for MoS_2_ and WS_2_ FETs. For a fair comparison, we project the scaled-*SS* (SSS) for an EOT of 0.9 nm using the *D*_IT_. We found SSS to be 76 mV.dec^−1^ and 80 mV.dec^−1^ for MoS_2_ and WS_2_, respectively, and 64 mV.dec^−1^ for the UTB Si MOSFET in ref. ^36^. A similar exercise was performed for other reports on MoS_2_ FETs from the literature and the results are summarized in Table 2. The impact of higher *D*_IT_ at the TMD/Al_2_O_3_ interface can be mitigated either by scaling the EOT (i.e., increasing *C*_OX_)^37^ or by improving the interface (i.e., reducing *D*_IT_). The presence of structural defects such as sulfur vacancies are known to introduce trap sites which contribute to *D*_IT_. It has been found that *D*_IT_ can be reduced by various surface passivation techniques^38,39^. In addition, photoresist residue from the lithography and/or the wet transfer process can cause an increase in *D*_IT_. Therefore it is possible to reduce *D*_IT_ through further optimization of growth, post-growth processing, and improvement in fabrication process flow.

**Table 2:** Benchmarking median subthreshold slope for *L*_CH_ = 100 nm.

	SS (mV.dec^−1^)	EOT (nm)	Gate dielectric	*D*_IT_ (10^12^ eV^−1^ cm^−2^)	SSS (mV.dec^−1^) at SEOT = 0.9 nm
^7^—MoS_2_	80	1.9	4 nm HfO_2_	3.7 × 10^12^	70
^7^—MoS_2_	160	2.7	8 nm HfO_2_	1.3 × 10^13^	93
^7^—MoS_2_	200	3.8	12 nm HfO_2_	1.3 × 10^13^	93
^7^—MoS_2_	1350	50	50 nm SiO_2_	9.2 × 10^12^	83
Our work-MoS_2_	450	22	50 nm Al_2_O_3_	6.3 × 10^12^	76
Our Work-WS_2_	550	22	50 nm Al_2_O_3_	8 × 10^12^	80
^36^—UTB SOI	80	4	4 nm SiO_2_	1.8 × 10^12^	64

#### Current ON/OFF ratio

Fig. 4g, h show the distribution of the ratio of maximum to minimum current (*I*_max_/*I*_min_) across all MoS_2_ and WS_2_ FETs, respectively. Here, *I*_max_ is the maximum current obtained from the transfer characteristics for *V*_DS_ = 1 V and *I*_min_ is the average noise floor. Note that the true device current in the OFF-state is beyond the measurement range of the instrument. See Supplementary Fig. 4a, b for the distribution of *I*_max_ and *I*_min_, Supplementary Fig. 4c, d for the distribution of *I*_max_/*I*_min_ for different *L*_CH_ for MoS_2_ and WS_2_ FETs, and Supplementary Table 3 for a summary of device-to-device variation in *I*_max_/*I*_min_. The median and standard deviation for *I*_max_/*I*_min_ were found to be 2.1 × 10^7^ and 5.5 × 10^7^ for MoS_2_ FETs and 2.1 × 10^7^ and 2.6 × 10^7^ for WS_2_ FETs. These values are over an order of magnitude higher than the *I*_max_/*I*_min_ of 1.3 × 10^6^ for UTB Si MOSFETs^36^. *I*_max_/*I*_min_ is benchmarked against literature reports for *L*_CH_ = 100 nm as shown in Supplementary Table 4. Note that the key OFF-state performance indicators, i.e., threshold voltage, SS, *D*_IT_, and *I*_max_/*I*_min_, are mostly found to be independent of *L*_CH_. Even for *L*_CH_ = 100 nm, no detrimental short-channel effects are observed, which is expected and can be ascribed to the atomically thin body nature of monolayer TMDs, as well as the use of thin and high-k Al_2_O_3_ as the gate dielectric with EOT = 22 nm.

#### Field-effect mobility and contact resistance

**Fig. 5:**
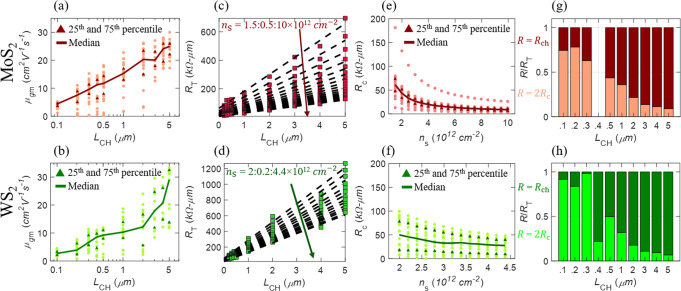
Device-to-device variation in field-effect mobility and contact resistance. Distribution of mobility extracted using peak transconductance ($\mu _{g_{\mathrm{m}}}$) for different channel lengths for **a** MoS_2_ and **b** WS_2_ FETs. Median, 25th percentile, and 75th percentile is also denoted. Total resistance (*R*_T_) versus *L*_CH_ for **c** MoS_2_ and **d** WS_2_ for different carrier concentrations (*n*_S_) extracted using a representative TLM structure. The distribution of contact resistance (*R*_c_) across multiple TLM structures, extracted from the y-intercepts in **c** and **d**, as a function of *n*_S_ for **e** MoS_2_ and **f** WS_2_, respectively. The relative contribution of *R*_c_ and channel resistance (*R*_ch_) to the total resistance for **g** MoS_2_ and **h** WS_2_ for different *L*_CH_. In scaled devices, as *R*_ch_ scales with the channel length, the contribution of *R*_c_ (note that *R*_c_ is independent of *L*_CH_), i.e., 2*R*_c_/*R*_T_, is more significant compared to *R*_ch_, i.e., *R*_ch_/*R*_T_.

Field-effect mobility (*μ*_FE_) is an important device parameter that strongly influences the ON-state performance of a FET. While intrinsic mobility is a material related parameter, *μ*_FE_ is determined by extrinsic effects, such as contact resistance (*R*_c_), and often depends on how it is extracted from the device characteristics. Three popular methods for extracting *μ*_*FE*_ are peak transconductance ($\mu _{g_{\mathrm{m}}}$), Y-function (*μ*_*Y*_)^40^, and TLM (*μ*_TLM_) as described in Supplementary Note 3. Figure 5a, b, show the distribution and the corresponding median values for $\mu _{g_{\mathrm{m}}}$ as a function of *L*_CH_ for MoS_2_ and WS_2_ FETs, respectively. Additionally, 25th and 75th percentile values of the distribution are also marked. Clearly, $\mu _{g_{\mathrm{m}}}$ shows a strong *L*_CH_ dependence, with the median value varying from 23.9 cm^2^ V^−1^ s^−1^ to 3.6 cm^2^ V^−1^ s^−1^ for MoS_2_ and 29 cm^2^ V^−1^ s^−1^ to 2.7 cm^2^ V^−1^ s^−1^ for WS_2_ as the devices are scaled from *L*_CH_ = 5 μm down to *L*_CH_ = 100 nm. Supplementary Fig. 5a, b shows a similar analysis of *μ*_*Y*_ for MoS_2_ and WS_2_ FETs, respectively and Supplementary Table 5 summarizes the device-to-device variation in $\mu _{g_{\mathrm{m}}}$ and *μ*_*Y*_. Both $\mu _{g_{\mathrm{m}}}$ and *μ*_*Y*_ extracted from shorter-channel devices show significant reduction in their median values, indicating the dominant role of *R*_c_ in scaled 2D FETs^6^. The contact resistance is seen as a result of Fermi-level pinning at the metal/TMD contact interface, resulting in a finite SB height^30^. To investigate further, we used the TLM structure shown in Fig. 2c to extract *R*_c_ and evaluate its impact on *L*_CH_ scaling as shown in Fig. 5c–f. We used Eq. (3) to extract *R*_c_.

3 $$	R_{\mathrm{T}} = 2R_{\mathrm{c}} + R_{{\mathrm{ch}}}; R_{{\mathrm{ch}}} = \frac{{L_{{\mathrm{CH}}}}}{{\mu _{{\mathrm{TLM}}}C_{{\mathrm{OX}}}(V_{{\mathrm{GS}}} - V_{{\mathrm{t}}_{{\mathrm{lin}}}})}} = \frac{{L_{{\mathrm{CH}}}}}{{qn_{\mathrm{S}}\mu _{{\mathrm{TLM}}}}}; \\ 	 n_{\mathrm{S}} = \frac{{C_{{\mathrm{OX}}}(V_{{\mathrm{GS}}} - V_{{\mathrm{t}}_{{\mathrm{lin}}}})}}{q}$$

Here, *R*_T_ is the total measured resistance of the FET, and *R*_ch_ is the channel resistance, which is directly proportional to *L*_CH_ and inversely proportional to the carrier density (*n*_S_) when the FET is measured in the linear operation regime. However, *R*_c_ is independent of *L*_CH_ and hence can be extracted from the y-intercept of *R*_T_ *versus L*_CH_ plots, as shown in Fig. 5c, d for MoS_2_ and WS_2_, respectively, for different *n*_S_^41^ (see Supplementary Note 4 for further discussion on the extraction of *n*_S_). Figure 5e, f show the distribution of corresponding extracted *R*_c_ as a function of *n*_S_. A steady decrease in *R*_c_ with increasing *n*_S_ is attributed to the phenomenon of contact-gating in global back-gated FET geometry, since the SB width at the metal/2D interface is modulated by the back-gate voltage^30^. Lower SB width allows for easier carrier tunneling, reducing *R*_c_. For the MoS_2_ FET, the median *R*_c_ value was found to be 9.2 kΩ−μm, corresponding to *n*_S_ = 1 × 10^13^ cm^−2^. However, for WS_2_, *n*_S_ was limited to 4.4 × 10^12^ cm^*−*2^, owing to the more positive $V_{{\mathrm{t}}_{{\mathrm{lin}}}}$, resulting in a higher median *R*_c_ = 29.2 kΩ−μm. For a case of identical carrier concentration, *n*_S_ = 2.7 × 10^12^ cm^*−*2^, similar median *R*_c_ values of 33 kΩ−μm and 39.4 kΩ−μm are obtained for MoS_2_ and WS_2_, respectively. The difference in *R*_c_ between MoS_2_ and WS_2_ can be explained from the fact that the charge neutrality level is closer to the conduction band for MoS_2_ than it is for WS_2_, resulting in a higher SB height at the Ni/WS_2_ contact interface compared to the Ni/MoS_2_ contact interface^42^.

The relative effect of *R*_c_ is assessed for different *L*_CH_. Figure 5g, h show the contribution of *R*_c_ and *R*_ch_ to the total resistance *R*_T_ using stacked bar plots as a function of *L*_CH_ for MoS_2_ and WS_2_, respectively. It is clear that for *L*_CH_ ≤ 1 μm, the contact effects are significant since 2*R*_c_ > *R*_ch_. This explains why the extracted $\mu _{g_{\mathrm{m}}}$ is *L*_CH_ dependent and is severely underestimated by more than 80% for both MoS_2_ and WS_2_ when extracted from scaled devices with *L*_CH_ = 100 nm. Since $\mu _{g_{\mathrm{m}}}$ extraction is limited by *R*_c_, extracting *μ*_TLM_ following Eq. (3) is more appropriate for short channel devices. Supplementary Fig. 5c, d show the distribution of *μ*_TLM_ across MoS_2_ and WS_2_ TLM structures, respectively, and Supplementary Table 6 summarizes the variation across the different TLM structures. The extracted median value for *μ*_TLM_ was found to be 27 cm^2^ V^−1^ s^−1^ and 16 cm^2^ V^−1^ s^−1^ for MoS_2_ and WS_2_ FETs, respectively. Long channel devices are less vulnerable to *R*_c_ and corresponding $\mu _{g_{\mathrm{m}}}$ values are more accurate representations of intrinsic channel mobility, albeit with some challenges as described by Nasr et al.^43^. Nevertheless, our “champion” long-channel MoS_2_ and WS_2_ FETs with *L*_CH_ = 5 μm demonstrated $\mu _{g_{\mathrm{m}}}$ = 30 cm^2^ V^−1^ s^−1^ and 33 cm^2^ V^−1^ s^−1^, respectively. Similarly, “champion” MoS_2_ and WS_2_ TLM structures demonstrated *μ*_TLM_ = 46 cm^2^ V^−1^ s^−1^ and 33 cm^2^ V^−1^ s^−1^, respectively.

Table 3 shows the benchmarking of our “champion” devices with the best reports from the literature using *μ*_FE_ ($\mu _{g_{\mathrm{m}}}$ for longer channel devices and *μ*_TLM_ for shorter channel devices) and *R*_c _for both MoS_2_ and WS_2_. We have also included median/mean values wherever applicable. Note that while higher *μ*_FE_ values have been reported based on “champion” exfoliated and CVD grown MoS_2_ FETs^7,12,24,25,44–46^, our report is statistically more significant as it demonstrates variation across multiple TLM structures. For WS_2_, *μ*_FE_ = 33 cm^2^ V^−1^ s^−1^ is the highest reported, 1.5X better than the previous report on synthetic WS_2_^47^. Higher *μ*_FE_ values reported for WS_2_ are either for exfoliated materials at room temperature^48^ and low temperatures^49^, or for CVD grown materials with contact engineering via the use of multilayer graphene as interlayers^50^. More interestingly, UTB Si MOSFETs with 0.9 nm thick Si show *μ*_FE_ ≈ 6 cm^2^ V^−1^ s^−1^^51^, which is more than 2 orders of magnitude smaller compared to bulk Si mobility and is primarily attributed to thickness fluctuation in UTB Si.

**Table 3:** Benchmarking ON-state performance at *V*_DS_ = 1 V (best values are compared with median/mean values shown within parentheses).

	*μ*(cm^2^ V^−1^ s^−1^)	*R*_c_(kΩ−μm)	*I*_ON_(μA.μm^−1^)	*n*_S_(cm^−2^)
^25^—MoS_2_	$\mu _{g_{\mathrm{m}}}$ = 42 (34.2)	0.73 (1)	22, *L*_CH_ = 5.4 μm	1.3 × 10^13^
^22^—MoS_2_	*μ*_TLM_ = 20	6.5	270, *L*_CH_ = 80 nm	1 × 10^13^
^12^—MoS_2_	$\mu _{g_{\mathrm{m}}}$ = 80 (≈40)	2.4	13, *L*_CH_ = 4 μm	6.6 × 10^12^
^65^—MoS_2_	*μ*_TLM_ = 30	1.7	260, *L*_CH_ = 10 nm	4.7 × 10^13^
^7^—MoS_2_	*μ*_TLM_ = 15	1	250, *L*_CH_ = 29 nm	1.5 × 10^13^
^26^—MoS_2_	*μ*_4-point_ ≈ 75 (70)	14	–	–
Our work-MoS_2_	*μ*_TLM_ = 47 (27)	3(9.2)	73 (54), *L*_CH_ = 100 nm	1 × 10^13^
^64^—WS_2_	$\mu _{g_{\mathrm{m}}}$ = 11	–	25, *L*_CH_ = 4 μm	2.1 × 10^13^
^47^—WS_2_	$\mu _{g_{\mathrm{m}}}$ = 20.4	–	0.6, *L*_CH_ = 1 μm	2.5 × 10^12^
^50^—WS_2_	$\mu _{g_{\mathrm{m}}}$ = 5	–	≈0.05, *L*_CH_ = 10 μm	≈7.2 × 10^12^
^50^—WS_2_ (Graphene contact)	$\mu _{g_{\mathrm{m}}}$ = 50 (27)	–	≈1.1, *L*_CH_ = 10 μm	≈7.2 × 10^12^
Our work-WS_2_	*μ*_TLM_ = 33 (16)	2.1 (29)	26 (17), *L*_CH_ = 100 nm	4.4 × 10^12^
^51^—UTB SOI	*μ*_4-point_ = 6	–	≈35 * 10^−3^, *L*_CH_ = 100 μm	≈9 × 10^12^

Metal/2D contact resistances are comparatively high even for the “champion” devices with *R*_c_ = 3 kΩ−μm and *R*_c_ = 2.1 kΩ−μm for MoS_2_ and WS_2_, respectively, when compared to the *R*_c_ = 0.1 kΩ−μm typically reported for state-of-the-art Si FETs. However, various methods have been developed to reduce the effect of SB-limited carrier transport in 2D TMDs^52^, such as work function engineering to reduce the SB height^30^, introduction of interlayers such as graphene to decouple the metal/2D interface to alleviate Fermi-level pinning^53,54^, and achieving higher carrier concentration underneath or near the metal/2D contacts through substitutional or surface charge transfer doping to reduce the SB width^42,55^. Nevertheless, our MOCVD grown monolayer MoS_2_ FETs demonstrate *R*_c_ similar to values reported in the literature^7,22,25,56^. The “champion” devices are benchmarked in Table 3. To the best of our knowledge, this is the first report of *R*_c_ for synthetic WS_2_. Additionally, our work marks the first study on the extraction of contact resistance from multiple TLM structures for both MoS_2_ and WS_2_. Smithe et al.^25^ have demonstrated a pseudo-TLM analysis where independent devices with different channel lengths and widths were used to extract the distribution of *R*_T_. TLM analysis is done on the devices between 10th and 90th percentile^25^. Our demonstration involves the extraction of contact resistance from separate TLM structures and finding the variation across these TLM structures, and the analysis is not limited to a percentile limit.

#### Drive-current and saturation velocity

**Fig. 6:**
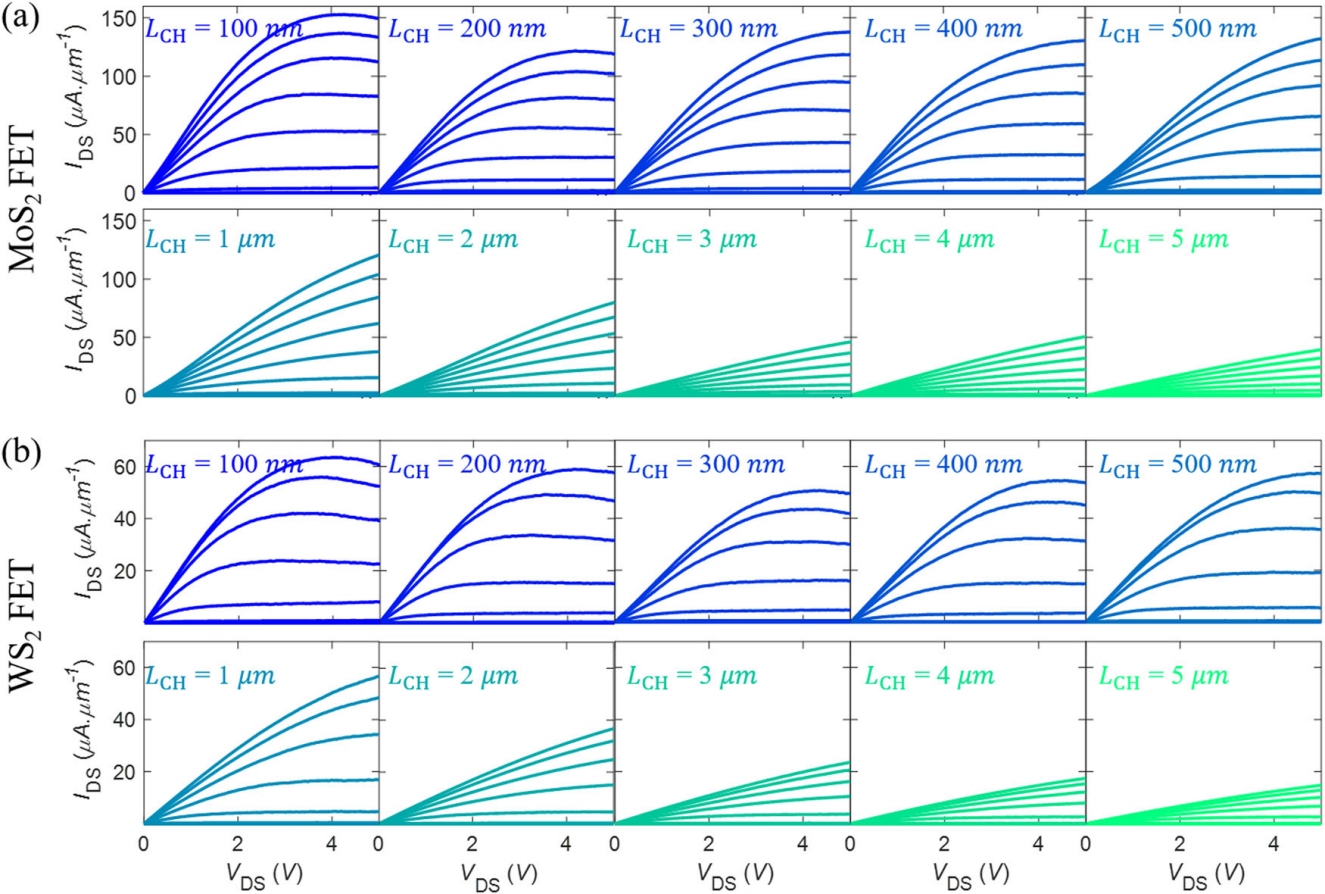
ON-state performance of monolayer MoS_2_ and WS_2_ FETs. Output characteristics for channel lengths ranging from *L*_CH_ = 100 nm to *L*_CH_ = 5 μm, obtained from a representative transmission-line measurement structure for **a** MoS_2_ and **b** WS_2_ FETs. Current saturation is achieved in shorter-channel devices for both MoS_2_ and WS_2_ FETs.

Finally, high performance FETs are benchmarked using the drive current (*I*_ON_) that is achievable for a given supply voltage (*V*_DS_ = *V*_DD_). Higher values of *I*_ON_ ensure faster circuit operation as the intrinsic delay of a FET is proportional to *CV*_DD_/*I*_ON_, where *C* is the load capacitance. In digital electronics, higher *I*_ON_ allows larger fan-out. Figure 6a, b display the output characteristics of MoS_2_ and WS_2_ FETs, respectively, for different channel lengths, which were used to assess the ON-state performance of the devices. At high biases, high current density leads to self-heating, resulting in negative differential resistance (NDR) behavior. This is a common phenomenon seen in ultra-thin body FETs, including SOI FETs^57^, nanowire FETs^58^, graphene FETs^59^, and, more recently, exfoliated multilayer MoS_2_ FETs^60^ and CVD grown monolayer MoS_2_ FETs^61^. It is possible to reduce or eliminate the self-heating effect through pulsed measurements with pulse widths less than 100 μs^60^.

**Fig. 7:**
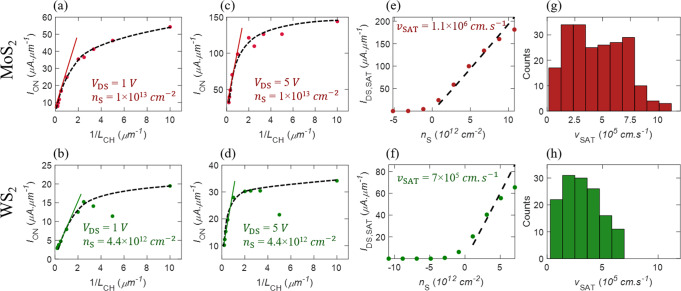
Drive current and saturation velocity. Median of drive current (*I*_ON_) as a function of 1/*L*_CH_ for **a** MoS_2_ and **b** WS_2_ FETs at *V*_DS_ of 1 V corresponding to the linear region of the FETs, and **c** MoS_2_ and **d** WS_2_ FETs at *V*_DS_ of 5 V corresponding to the saturation region of the FETs. These are extracted at carrier concentrations (*n*_S_) of 1 × 10^13^ cm^−2^ and 4.4 × 10^12^ cm^−2^ for MoS_2_ and WS_2_, respectively. The saturation current (*I*_DS,SAT_) for **e** MoS_2_ and **f** WS_2_, extracted from their corresponding shorter-channel devices (*L*_CH_ < 1 μm) as a function of *n*_S_. The slope indicates the saturation velocity (*v*_SAT_). The distribution of *v*_SAT_ for **g** MoS_2_ and **h** WS_2_ shorter-channel FETs.

Figure 7a–d show the median for *I*_ON_ as a function of *L*_CH_ for *V*_DS_ = 1 V and *V*_DS_ = 5 V for MoS_2_ and WS_2_ FETs, respectively, extracted from their respective output characteristics. For both TMDs, at low *V*_DS_ = 1 V, i.e., in the linear region, *I*_ON_ is expected to demonstrate an inverse channel length dependence following Eq. (4).

4 $$\frac{{I_{{\mathrm{ON}}}}}{W} = \frac{{I_{{\mathrm{DS}},{\mathrm{LIN}}}}}{W} = qn_{\mathrm{S}}\mu _{g_{\mathrm{m}}}\frac{{V_{{\mathrm{DS}}}}}{{L_{{\mathrm{CH}}}}}$$

This trend is observed for both MoS_2_ and WS_2_ FETs in Fig. 7a, b, respectively, for channel lengths *L*_CH_ ≥ 1 μm. However, for devices with channel length *L*_CH_ < 1 μm, the inverse channel length dependence is obscured by *R*_c_. Similar linear dependence is observed for *I*_ON_ in longer-channel devices (*L*_CH_ ≥ 1 μm) at *V*_DS_ = 5 V for both MoS_2_ and WS_2_ FETs in Fig. 7c, d, respectively, following Eq. (5).

5 $$\frac{{I_{{\mathrm{ON}}}}}{W} = \frac{{I_{{\mathrm{DS}},{\mathrm{SAT}}}}}{W} = \frac{{C_{{\mathrm{OX}}}\mu _{g_{\mathrm{m}}}(V_{{\mathrm{GS}}} - V_{{\mathrm{t}}_{{\mathrm{lin}}}})^2}}{{2L_{{\mathrm{CH}}}}} = \frac{{q^2\mu _{g_{\mathrm{m}}}}}{{C_{{\mathrm{OX}}}}}\frac{{n_{\mathrm{S}}^2}}{{2L_{{\mathrm{CH}}}}}$$

These results are in accordance with classic long-channel FET characteristics (i.e., at low drain bias, the device operates in the linear regime (Eq. (4)), whereas for $V_{{\mathrm{DS}}} \ge V_{{\mathrm{GS}}} - V_{{\mathrm{t}}_{{\mathrm{lin}}}}$, the channel is pinched-off, resulting in current saturation). The saturation current follows a square-law dependence on the overdrive voltage and, therefore, on *n*_S_ (Eq. (6))^62^. In shorter-channel devices (*L*_CH_ < 1 μm), as the lateral electric field ($\xi \approx \frac{{V_{{\mathrm{DS}}}}}{{L_{{\mathrm{CH}}}}}$) becomes more than the critical electric field (*E*_C_), the carrier velocity reaches saturation velocity (*v*_SAT_). This leads to current saturation, with the saturation current being independent of *L*_CH_ as described by Eq. (6)^62^.

6 $$\frac{{I_{{\mathrm{ON}}}}}{W} = \frac{{I_{{\mathrm{DS}},{\mathrm{SAT}}}}}{W} = C_{{\mathrm{OX}}}v_{{\mathrm{SAT}}}\left( {V_{{\mathrm{GS}}} - V_{{\mathrm{t}}_{{\mathrm{lin}}}}} \right) = qn_{\mathrm{S}}v_{{\mathrm{SAT}}}$$

However, in order to observe current saturation due to velocity saturation, the drain bias must meet the criterion given by Eq. (7).

7 $$L_{{\mathrm{CH}}}\frac{{v_{{\mathrm{SAT}}}}}{{\mu _{g_{\mathrm{m}}}}} \,<\, V_{{\mathrm{DS}}} \,<\, \left( {V_{{\mathrm{GS}}} - V_{{\mathrm{t}}_{{\mathrm{lin}}}} = \frac{{qn_{\mathrm{S}}}}{{C_{{\mathrm{OX}}}}}} \right)$$

For example, as seen in Fig. 6, current saturation is achieved at *V*_DS_ = 4 V for 100 nm MoS_2_ FET and WS_2_ FET, which is much lower than the corresponding $V_{{\mathrm{GS}}} - V_{{\mathrm{t}}_{{\mathrm{lin}}}}$ of 11.6 V and 6.7 V, respectively. This explains why the drive current in shorter-channel MoS_2_ and WS_2_ FETs display little-to-no channel length dependence for high drain bias (*V*_DS_ = 5 V), as seen in Fig. 7c, d. Nevertheless, scaled MoS_2_ and WS_2_ FETs with channel lengths of 100 nm demonstrate high median drive currents of *I*_ON_ = 54 μA.μm^−1^ and *I*_ON_ = 17 μA.μm^−1^, respectively, for *V*_DS_ = 1 V and *I*_ON_ = 146 μA.μm^−1^ and *I*_ON_ = 30 μA.μm^−1^, respectively, for *V*_DS_ = 5 V. Furthermore, *I*_ON_ at *V*_DS_ = 5 V can reach as high as 161 μA.μm^−1^ and 53 μA.μm^−1^ in “champion” MoS_2_ and WS_2_ FETs, respectively. The distribution of *I*_ON_ for *V*_DS_ = 1 V and *V*_DS_ = 5 V as a function of *L*_CH_ is shown in Supplementary Fig. 6 and the corresponding device-to-device variation is summarized in Supplementary Table 7 for MoS_2_ and WS_2_ FETs. The higher drive current seen for MoS_2_ FETs compared to that of WS_2_ FETs is a direct consequence of lower $V_{{\mathrm{t}}_{{\mathrm{lin}}}}$, which allows for higher *n*_S_ in MoS_2_ channels. Further improvement in the drive current of scaled 2D FETs can be achieved by reducing *R*_c_. Note that, while there are reports of higher *I*_ON_ in large-area grown MoS_2_ films, none of the earlier studies provide extensive device statistics^22,63–65^. *I*_ON_ for UTB Si MOSFET is 35 nA.μm^−1^ for 0.9 nm thick Si^51^. The “champion” devices are benchmarked in Table 3. Supplementary Table 8 shows benchmarking of our statistical study on MoS_2_ FETs using field-effect mobility and drive current (at *V*_DS_ = 2 V) with similar channel length dependent statistical studies from the literature. The mean and standard deviation is compared with the *L*_CH_ dependence and plotted in Supplementary Fig. 7. Better performance is seen for our channel length dependence study compared to ref. ^66^ for both the drive current and mobility.

Finally, saturation velocity (*v*_SAT_) is another key material parameter that determines *I*_ON_ in scaled FETs. This is because at low lateral electric field (*ξ*) the average electron drift velocity increases linearly through the mobility ($v_{\mathrm{d}} = \mu _{{\mathrm{FE}}}\xi$), but at large electric fields, which are easily achievable in sub-micron FETs, the carrier velocity saturates. Thus, *I*_ON_ becomes less dependent on *μ*_FE_ and is instead proportional to *v*_SAT_, following Eq. (6). Additionally, high *v*_SAT_ is needed for faster switching^11^. Figure 7e–h show the extraction of *v*_SAT_ and the distribution of *v*_SAT_ for MoS_2_ and WS_2_, respectively. The linear dependence of the saturation current (*I*_DS,SAT_) on *n*_S_ following Eq. (6), is used to extract *v*_SAT_. Median *v*_SAT_ values of 6.4 × 10^5^ cm.s^−1^ and 4 × 10^5^ cm.s^−1^ and “champion” *v*_SAT_ values of 1.1 × 10^6^ cm.s^−1^ and 6.9 × 10^5^ cm.s^−1^ are obtained for MoS_2_ and WS_2_, respectively. The corresponding device-to-device variations are summarized in Supplementary Table 9. The *v*_SAT_ values are significantly lower compared to bulk Si with *v*_SAT_ ≈ 10^7^ cm.s^−1^^67,68^. Nathawat et al. have reported higher *v*_SAT_ ≈ 6 × 10^6^ cm.s^−1^ in CVD grown monolayer MoS_2_^69^. However, their measurements were done using nanosecond range pulses to reduce the impact of self-heating and hot carrier capture by deep oxide traps. For WS_2_, this is the first report of *v*_SAT_.

## Discussion

In conclusion, we have performed a detailed study of device-to-device variation and impact of channel length scaling on the electrical parameters, such as threshold voltage, subthreshold slope, density of interface trap states, ratio of minimum to maximum current, field-effect electron mobility, drive current, contact resistance, and saturation velocity, of MOCVD grown MoS_2_ and WS_2_ monolayer based FETs using statistical measures such as median, mean, standard deviation, and minimum/maximum values and have benchmarked our findings against other similar reports from 2D literature as well as UTB Si FETs. While in absolute terms the spatial variations in the respective benchmarking parameters appear to be large for MoS_2_ and WS_2_ FETs, when compared at scaled oxide thickness, our results are not significantly different from the projected variations for UTB Si FETs. Our “champion” long-channel MoS_2_ and WS_2_ FETs with *L*_CH_ = 5 μm demonstrated electron mobilities of 30 cm^2^ V^−1^ s^−1^ and 33 cm^2^ V^−1^ s^−1^, respectively, when extracted using peak transconductance and 46 cm^2^ V^−1^ s^−1^ and 33 cm^2^ V^−1^ s^−1^, respectively, when extracted using TLM method. For synthetic monolayer WS_2_ films, these are the highest reported room temperature electron mobilities, 1.5X better than the best report from the literature. Similarly, our “champion” shortest channel length MoS_2_ and WS_2_ FETs, with *L*_CH_ = 100 nm, demonstrated drive currents as high as 161 μA.μm^−1^ and 53 μA.μm^−1^ for *V*_DS_ = 5 V at carrier densities of *n*_S_ = 1 × 10^13^ cm^−2^ and 4.4 × 10^12^ cm^−2^, respectively, in spite of the presence of high contact resistances. We attribute our accomplishments to the epitaxial growth of highly crystalline 2D monolayers on sapphire substrate *via* MOCVD at 1000 °C using chalcogen and sulfur precursors that minimize carbon contamination in the film, as well as to the clean transfer of the film from the growth substrate to the device fabrication substrate. Our findings suggest that 2D FETs are promising alternatives for future VLSI circuits.

## Methods

### MOCVD growth

Uniform monolayer deposition was achieved in a cold-wall horizontal reactor with an inductively heated graphite susceptor equipped with wafer rotation as previously described^27^. Molybdenum hexacarbonyl (Mo(CO)_6_) and tungsten hexacarbonyl (W(CO)_6_) were used as metal precursors while hydrogen sulfide (H_2_S) was the chalcogen source with H_2_ as the carrier gas. Mo(CO)_6_ maintained at 10 °C and 950 Torr in a stainless-steel bubbler was used to deliver 0.036 *sccm*. W(CO)_6_ maintained in a bubbler at 10 °C and 760 Torr delivered 6.4 × 10^−4^ sccm. The flow rate of H_2_S was 400 sccm and the reactor pressure was 50 Torr for both sulfides. MoS_2_ was deposited in a single step process at 1000 °C where coalesced monolayer growth across the 2″ wafer was achieved in 18 min. WS_2_ was deposited using a multi-step process with nucleation at 850 °C and lateral growth at 1000 °C, which resulted in coalesced monolayer growth across the 2″ wafer in 10 min^28^. In both cases, after growth, the substrate was cooled in H_2_S to 300 °C to inhibit decomposition of the MoS_2_ and WS_2_ films.

### Material characterization

A Bruker Icon atomic force microscope was used to measure surface morphology and film thickness. Scanasyst AFM tips with a nominal tip radius of ≈2 nm and spring constant of 0.4 Nm^−1^ were used in the peak-force tapping mode for the measurements. Photoluminescence (PL) maps were acquired over a 5 × 5 μm^2^ area with a laser wavelength of 532 nm and 300 grooves per mm grating in a WITec apyron Confocal Raman Microscope. A PANalytical MRD diffractometer with a 5-axis cradle was used for in-plane X-ray diffraction characterization of the sulfide films^70^. A Cu anode X-ray tube operated at 40 kV accelerating voltage and 45 mA filament current was used as the X-ray source. On the primary beam side, a mirror with ¼° slit and Ni filter were used to filter the Cu Kα line. On the diffracted beam side, an 0.27° parallel plate collimator with 0.04 rad Soller slits with PIXcell detector in open detector mode were employed. To determine the in-plane epitaxial relation of the film with respect to a substrate, sample surface was ≈2–4° away from the X-ray incidence plane.

### Transfer of monolayer films

Both the MoS_2_ and WS_2_ films were grown on 2″ sapphire wafers. The 2″ sapphire wafers were then cut into 1 × 1 cm^2^ pieces. For each material, two (2) 1 × 1 cm^2^ sapphire substrates were chosen, one corresponding to the center and another one corresponding to the edge of the 2-inch wafer. To fabricate the FETs, monolayer MoS_2_ and WS_2_ films grown on sapphire substrates were transferred onto 1 × 1 cm^2^ device fabrication substrates, i.e., 50 nm Al_2_O_3_ on Pt/TiN/p^++^-Si, using a PMMA (polymethyl-methacrylate) -assisted wet transfer process. First, the sapphire substrate with the monolayer film was spin coated with PMMA and then baked at 180 °C for 90 s. The corners of the spin coated films were scratched using a razor blade and immersed inside a 1 M NaOH solution kept at 90 °C. Capillary action drew NaOH into the substrate/film interface, separating the PMMA/monolayer film stack from the sapphire substrate. The separated film was then rinsed multiple times inside a water bath and finally transferred onto the 50 nm alumina substrate and baked at 50 °C and 70 °C for 10 min each to remove moisture and residual PMMA, ensuring a pristine interface.

### Gate dielectric fabrication

Direct replacement of thermally oxidized SiO_2_ with a high-κ dielectric such as Al_2_O_3_ grown *via* atomic layer deposition (ALD) is a logical choice to scale the EOT. However, we found that a Al_2_O_3_/p^++^-Si interface is not ideal for back gated FET fabrication owing to higher gate leakage current, more interface trap states, and large hysteresis, all of which negatively impact the performance of the device. Replacing Si with Pt, a large work function metal (5.6 eV) allows for minimal hysteresis and trap state effects^71^. Since Pt readily forms a Pt silicide at temperatures as low as 300 °C, a 20 nm TiN diffusion barrier deposited by reactive sputtering was placed between the p^++^ Si and the Pt, permitting subsequent high temperature processing^72^. This conductive TiN diffusion barrier allows the back-gate voltage to be applied to the substrate, thus simplifying the fabrication and measurement procedures. The polycrystalline Pt introduces very little surface roughness to the final Al_2_O_3_ surface, with a rms roughness of 0.7 nm.

### Device fabrication

Back gated field-effect transistors (FET) are fabricated using e-beam lithography. To define the channel region the substrate is spin coated with PMMA and baked at 180 °C for 90 s. The photoresist is then exposed to e-beam and developed using 1:1 mixture of 4-methyl-2-pentanone (MIBK) and 2 propanol (IPA). The monolayer MoS_2_ film is subsequently etched using sulfur hexafluoride (SF_6_) at 5 °C for 30 s. Next the sample is rinsed in acetone and IPA to remove the photoresist. In order to fabricate the source/drain contacts the substrate is again spin coated with MMA and PMMA followed by the e-beam lithography, developed using MIBK and IPA, and e-beam evaporation of 40 nm Ni/30 nm Au stack. Finally, the photoresist is rinsed away by lift off process using acetone and IPA.

### Electrical characterization

Lake Shore CRX-VF probe station and Keysight B1500A parameter analyzer were used to perform the electrical characterization at room temperature in high vacuum (≈10^−6^ Torr). Standard DC sweeps are used for the measurements of transfer and output characteristics of all devices. To ensure that the FETs are stabilized, they are conditioned by multiple repetitions of the same measurement. The transfer characteristics are measured three times to condition each FET and the fourth measurement is used for the analysis. The output characteristics are measured twice following the transfer characteristics and the second measurement is used for the analysis. We have found that no burn-in procedure is needed to ensure proper contact formation. Both MoS_2_ and WS_2_ FETs were measured as-fabricated.

### Reporting summary

Further information on research design is available in the Nature Research Reporting Summary linked to this article.

## Supplementary information

Supplementary Information

Peer Review File

Solar Cells Reporting Summary

Reporting Summary

## Data Availability

The datasets generated during and/or analyzed during the current study are available from the corresponding authors on reasonable request.

## Supplementary information

**Supplementary information** The online version contains supplementary material available at 10.1038/s41467-020-20732-w.

## Code availability

The codes used for plotting the data are available from the corresponding authors on reasonable request.
