# Supplementary material for: Benchmarking monolayer MoS2 and WS2 field-effect transistors
Source: Nat Commun. 2021 Jan 29;12:693. doi: 10.1038/s41467-020-20732-w (PMC7846590; doi:10.1038/s41467-020-20732-w)
Supplement: Supplementary file 1 — Supplementary Information [file 41467_2020_20732_MOESM1_ESM.pdf]

Supplementary Information

Benchmarking Monolayer MoS_2 and WS_2 Field-Effect Transistors

Amritanand Sebastian[1], Rahul Pendurthi[1], Tanushree H Choudhury[2], Joan M Redwing[2,3,4] & Saptarshi Das[1,3,4]*

[1]*Deparment of Engineering Science and Mechanics, Pennsylvania State University, University Park, PA 16802*

[2]*2D Crystal Consortium-Materials Innovation Platform (2DCC-MIP), Pennsylvania State University, University Park, PA, 16802, USA*

[3]*Department of Materials Science and Engineering, Pennsylvania State University, University Park, PA 16802*

[4]*Materials Research Institute, Pennsylvania State University, University Park, PA 16802*

Supplementary Figure 1

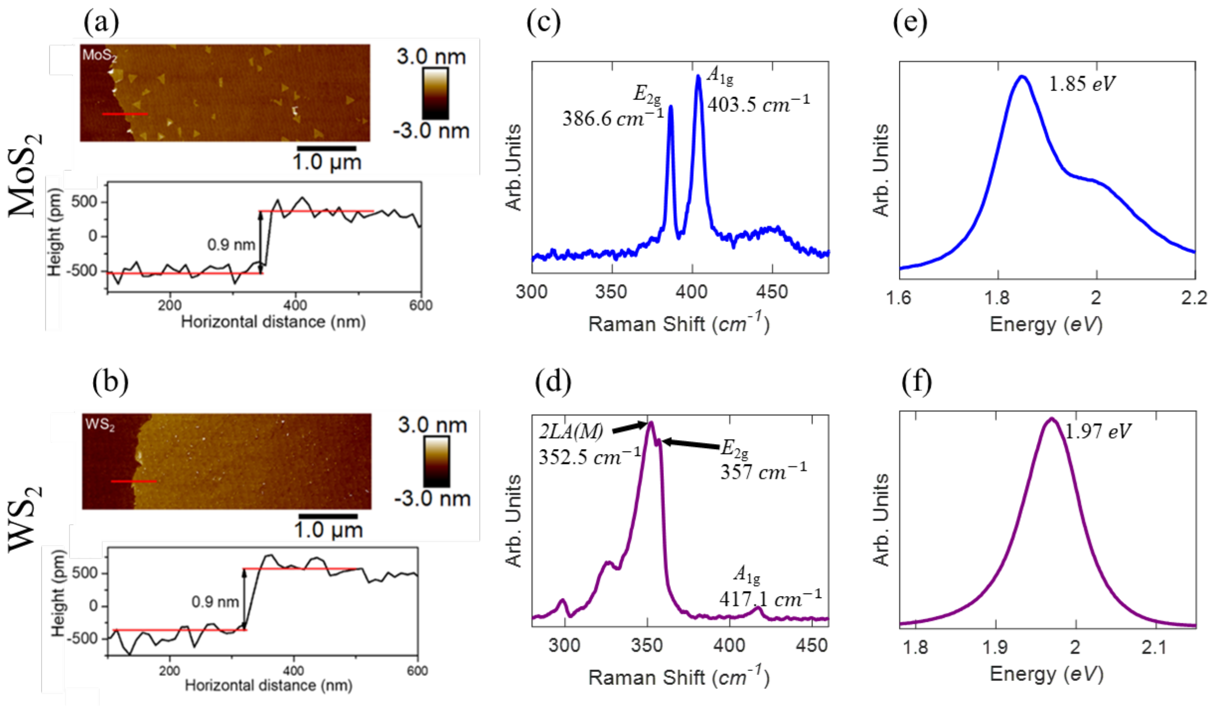

Supplementary Figure 1. Characterization of MOCVD grown monolayer WS_2 and MoS_2. AFM of scratch and height profile across the scratch along the red line for (a) MoS_2 and (b) WS_2. Raman spectrum with the characteristic peaks for c) MoS_2 with E_{2g} at 386.6 cm^{-1} and A_{1g} at 403.5 cm^{-1} and d) WS_2 with E_{2g} at 357 cm^{-1}, A_{1g} at 417.1 cm^{-1} and 2LA(M) at 352.5 cm^{-1}. PL spectrum for e) MoS_2 and f) WS_2 showing the characteristic monolayer response with peaks at 1.85 eV and 1.97 eV, respectively.

Supplementary Figure 2

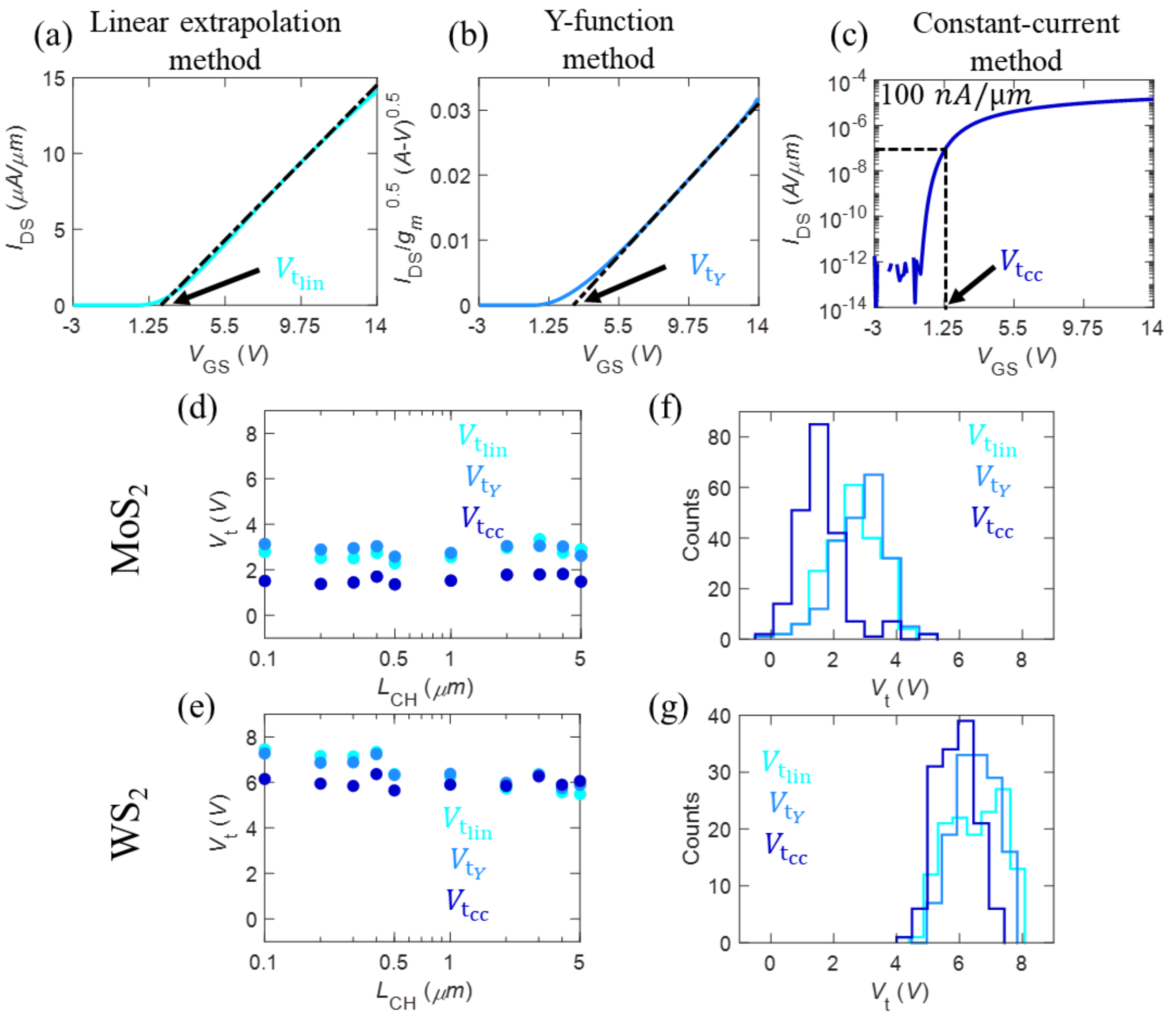

Supplementary Figure 2. Threshold voltage extraction, channel length dependence, and device to device variation. Threshold voltage extraction using a) linear extrapolation ($V_{t_{lin}}$), b) Y-function (V_{t_Y}), and c) constant-current method ($V_{t_{cc}}$). The median values for $V_{t_{lin}}$, V_{t_Y}, and $V_{t_{cc}}$ verses channel length (L_{CH}) showing no scaling effects for d) MoS_2 and e) WS_2. Histograms showing the variation in $V_{t_{lin}}$, V_{t_Y}, and $V_{t_{cc}}$ across all measured devices for f) MoS_2 and g) WS_2 FETs for different channel lengths.

Supplementary Note 1

In 2D FET literature, threshold voltage is extracted using various methods such as linear extrapolation ($V_{t_{lin}}$), Y-function (V_{t_Y}), and constant-current method ($V_{t_{cc}}$) as shown in Supplementary Fig. 2a-c. Linear extrapolation is the most common among these techniques. However, poor ON-state performance, presence of SB at the metal/semiconductor interface, and contact-gating effect in a back-gated geometry can limit the use of linear extrapolation. Y-function method is more appropriate for contact dominated FETs. Finally, constant current method is simply another threshold voltage extraction technique, which is discussed for completeness. Clearly none of the extracted threshold voltages, $V_{t_{lin}}$, V_{t_Y}, and $V_{t_{cc}}$ show any channel length (L_{CH}) dependence as evident from Supplementary Fig. 2d and 2e for MoS_2 and WS_2, respectively. Supplementary Fig. 2f and 2g show the distributions of $V_{t_{lin}}$, V_{t_Y}, and $V_{t_{cc}}$ for all measured MoS_2 and WS_2 FETs, respectively. For MoS_2 FETs, the distributions of V_{t_Y} and $V_{t_{lin}}$ are very similar with median values of 2.9 V and 2.5 V, respectively. However, $V_{t_{cc}}$ results in lower median value of 1.5 V for MoS_2, as the extraction is done at a constant current of 100 $nA/\mu m$, which is lower than the current at $V_{t_{lin}}$ and V_{t_Y}. A similar trend is observed for WS_2, where both V_{t_Y} and $V_{t_{lin}}$ have median values of 6.5 V and 6.4 V, while $V_{t_{cc}}$ has a median value of 5.9 V. The corresponding standard deviations were found to be 0.8 V for $V_{t_{lin}}$, V_{t_Y}, and $V_{t_{cc}}$ for MoS_2 and 0.8 V, 0.7 V, and 0.6 V, respectively, for $V_{t_{lin}}$, V_{t_Y}, and $V_{t_{cc}}$, in case of WS_2. Supplementary Table 1 summarize the statistics of $V_{t_{lin}}$, V_{t_Y}, and $V_{t_{cc}}$

Supplementary Table 1

Device-to-device variation in threshold voltage				
	MoS_2		WS_2	
	Median, Mean ± SD	Min, Max	Median, Mean ± SD	Min, Max
$V_{t_{cc}}$ (V)	1.5, 1.7 ± 0.8	-0.5, 5.3	5.9, 5.8 ± 0.6	4.2, 7.3
V_{t_Y} (V)	2.9, 2.8 ± 0.8	-0.1, 5.3	6.5, 6.5 ± 0.7	4.2, 7.8
$V_{t_{lin}}$ (V)	2.5, 2.9 ± 0.8	-0.1, 4.5	6.4, 6.5 ± 0.8	4.5, 8

Supplementary Note 2

It is important to discuss the possible origin of the variation seen in the electrical characteristics of monolayer MoS_2 and WS_2 FETs. Low device-to-device variation in MoS_2 and WS_2 FETs is attributed to the MOCVD growth of single-crystalline and epitaxial monolayers on the sapphire substrates. This is confirmed by in-plane XRD phi-scans in Fig. 1e and 1f, for MoS_2 and WS_2, respectively, which show six-fold rotational symmetry and epitaxial alignment of the monolayer with the underlying sapphire. If the films were polycrystalline with a high degree of misorientation within the plane of the film, then we would expect to see multiple peaks at different angles in the in-plane XRD scan. These films were also transferred to a Quantifoil Cu grid to investigate the microstructure in TEM by using selected area diffraction pattern (SAED) and dark field imaging. As shown in detailed materials characterization of these films (https://www.mri.psu.edu/2d-crystal-consortium/user-facilities/thin-films/list-thin-film-samples-available) the respective SAED patterns show a single crystalline pattern, while composite dark field maps illuminate two contrasting regions in the monolayer films. For MoS_2 these regions correspond to anti-phase domains. For WS_2 however, the regions are unidirectional and are separated by translational boundaries. We believe that it is possible to reduce the spatial variations in 2D FETs through further optimization of growth and improvement in fabrication process flow, which is unlikely for UTB Si owing to significant thickness variations at length scales similar to monolayer MoS_2 and WS_2. In addition, random dopant fluctuations and detrimental quantum confinement effects leading to increase in the bandgap of ultra-thin Si open up opportunities for 2D materials for advanced scaling nodes.

Supplementary Figure 3

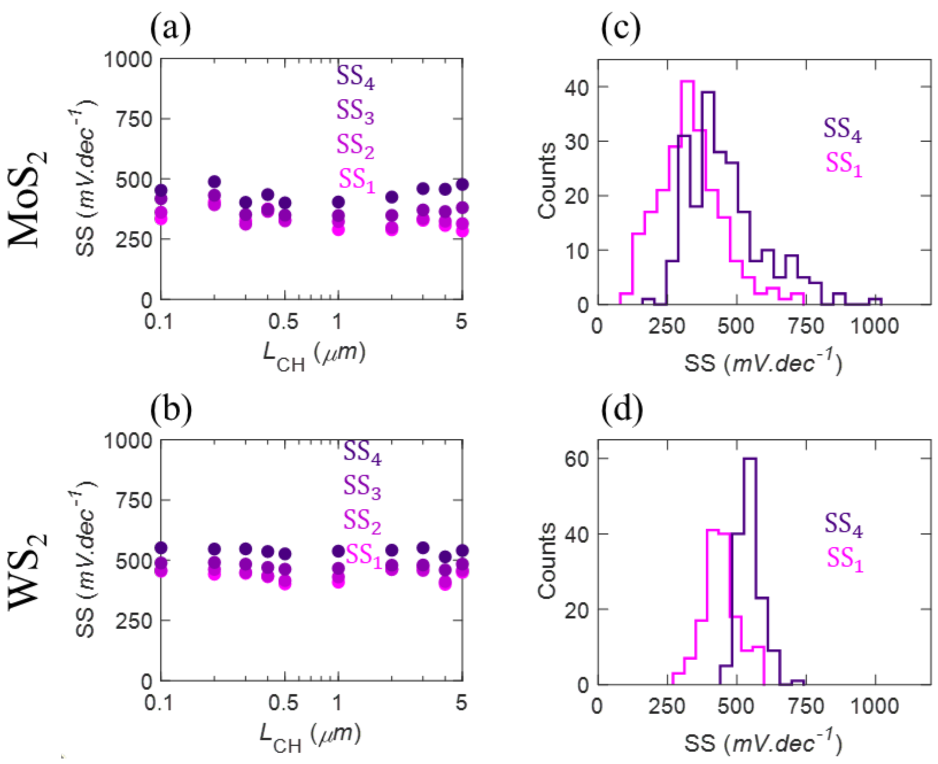

Supplementary Figure 3. Channel length dependence and device to device variation in subthreshold slope. The median subthreshold slope extracted over 1 (SS_1), 2 (SS_2), 3 (SS_3), and 4 (SS_4) orders of magnitude change in the drain current for a) MoS_2 and b) WS_2 as a function of L_{CH}. No L_{CH} dependence is observed. Histograms showing the device-to-device variation in SS_1 and SS_4 across all c) MoS_2 and d) WS_2 FETs.

Supplementary Table 2

Device-to-device variation in subthreshold slope and interface traps				
	MoS_2		WS_2	
	Median, Mean ± SD	Min, Max	Median, Mean ± SD	Min, Max
SS_1 $(mV.dec^{-1})$	327, 335 ± 115	93, 729	438, 443 ± 66	296, 596
SS_2 $(mV.dec^{-1})$	335, 344 ± 109	103, 740	449, 453 ± 56	305, 618
SS_3 $(mV.dec^{-1})$	369, 386 ± 116	124, 791	480, 483 ± 45	369, 621
SS_4 $(mV.dec^{-1})$	432, 460 ± 138	166, 1000	541, 546 ± 42	453, 738
D_{IT} $(10^{12}\ eV^{-1}cm^{-2})$	6.2, 6.6 ± 2.3	1.75, 15.7	8, 8.1 ± 0.7	6.5, 11.2

Supplementary Figure 4

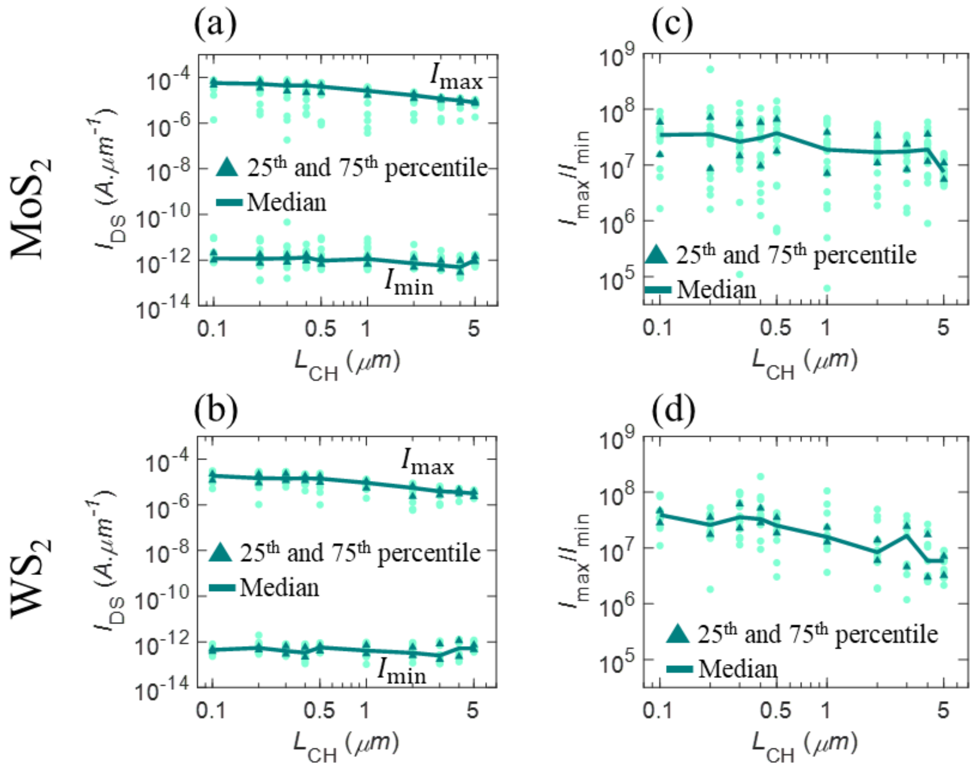

Supplementary Figure 4. Channel length dependence of maximum, minimum, and ratio of maximum to minimum current. Distribution of maximum current (I_{max}) and minimum current (I_{min}) for different channel lengths for a) MoS_2 and b) WS_2 FETs, extracted from the transfer characteristics. Median, 25th percentile and 75th percentile is denoted. Distribution of ratio of maximum to minimum current ratio (I_{max}/I_{min}) for different L_{CH} for c) MoS_2 and d) WS_2 FETs. I_{max}/I_{min} is mostly found to be independent of L_{CH} for both MoS_2 and WS_2 FETs. Note that I_{max} demonstrates some L_{CH} dependence for longer channel devices with $L_{CH} \geq 1$ μm for MoS_2 and WS_2 FETs due to the linear scaling law but can be ignored for I_{max}/I_{min} which is measured in orders of magnitude.

Supplementary Table 3

Device-to-device variation in I_{max}/I_{min}				
	MoS_2		WS_2	
	Median, Mean ± SD	Min, Max	Median, Mean ± SD	Min, Max
I_{max}/I_{min} (× 10^7)	2.1, 3.4 ± 5.5	6.2 × 10^{-3}, 52	2.1, 2.7 ± 2.6	0.1, 19

Supplementary Table 4

Benchmarking median $I_{\max}/I_{\min}$ for L_{CH} = 100 nm				
	$I_{\max}/I_{\min}$	EOT (nm)	$V_{\mathrm{GS,Range}}$ (V)	$SV_{\mathrm{GS,Range}}$ (V) at SEOT = 0.9 nm
[1] - MoS_2	≈7 × 10^6	1.9	1.5 to -0.5 (2)	0.94
[1] - MoS_2	≈4 × 10^6	50	-	-
Our Work - MoS_2	3.5 × 10^7	22	14 to -3 (17)	0.65
Our Work - WS_2	3.9 × 10^7	22	12 to -5 (17)	0.65
[2] - UTB SOI	1.3 × 10^6	4	1.8 to -0.2 (2)	0.45

Supplementary Note 3

Mobility from peak Transconductance: The field-effect electron mobility is extracted from the transconductance (g_{m}) using Supplementary Equation (S1).

$$\mu_{g_m} = \frac{dI_{\mathrm{DS}}}{dV_{\mathrm{GS}}}\left(\frac{L_{\mathrm{CH}}}{WC_{\mathrm{OX}}V_{\mathrm{DS}}}\right) \qquad (S1)$$

Y-function mobility: The linear part of Y-function, given by Supplementary Equation (S2) is extrapolated to obtain Y-function threshold. μ_Y is extracted using the slope of Y-function *versus* $V_{\mathrm{GS}} - V_{\mathrm{t}_Y}$

$$Y = \frac{I_{\mathrm{DS}}}{\sqrt{g_{\mathrm{m}}}} = (\mu_Y W C_{\mathrm{OX}} V_{\mathrm{DS}})^{0.5}(V_{\mathrm{GS}} - V_{\mathrm{t}_Y}) \qquad (S2)$$

TLM mobility: TLM mobility (μ_{TLM}) is obtained from the slope of the total resistance (R_{T}) *versus* L_{CH} using Supplementary Equation (S3).

$$R_{\mathrm{T}} = 2R_{\mathrm{c}} + R_{\mathrm{ch}};\ R_{\mathrm{ch}} = \frac{L_{\mathrm{CH}}}{\mu_{\mathrm{TLM}} C_{\mathrm{OX}}(V_{\mathrm{GS}} - V_{\mathrm{t_{lin}}})} = \frac{L_{\mathrm{CH}}}{q n_{\mathrm{S}} \mu_{\mathrm{TLM}}};\ n_{\mathrm{S}} = \frac{C_{\mathrm{OX}}(V_{\mathrm{GS}} - V_{\mathrm{t_{lin}}})}{q} \qquad (S3)$$

Supplementary Figure 5

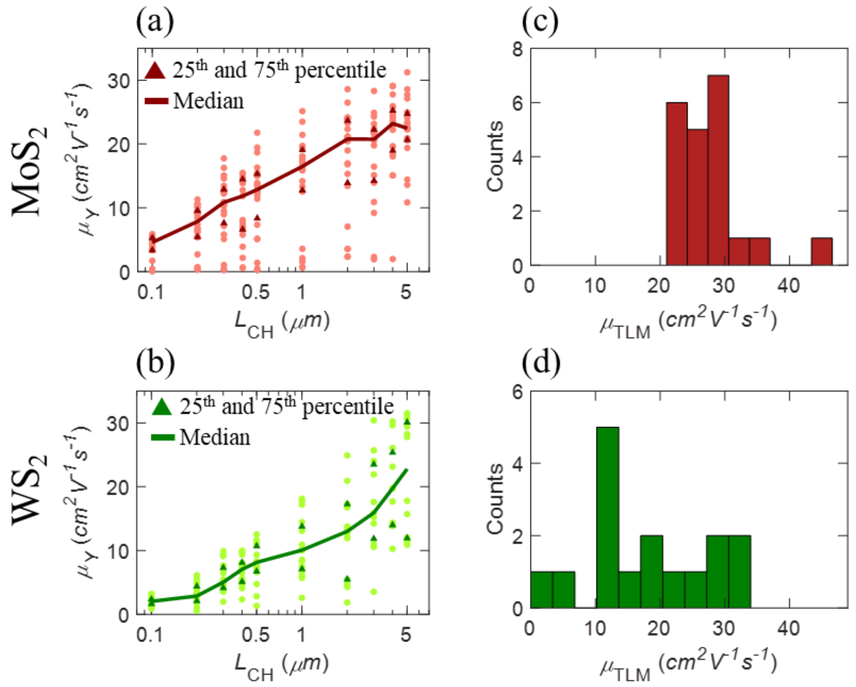

Supplementary Figure 5. Device-to-device variation in Y-function mobility and TLM mobility. The distribution of Y-function mobility (μ_Y) for different channel lengths (L_{CH}) for a) MoS_2 and b) WS_2 FETs. Median, 25th percentile and 75th percentile is denoted. The distribution of the TLM mobility (μ_{TLM}) for c) MoS_2 and d) WS_2

Supplementary Table 5

Device-to-device variation in field-effect carrier mobility								
	$\mu_{g_m}(cm^2\,V^{-1}s^{-1})$				$\mu_Y\ (cm^2\,V^{-1}s^{-1})$			
	MoS_2		WS_2		MoS_2		WS_2	
$L_{\mathrm{CH}}(\mu m)$	Median, Mean ± SD	Min, Max	Median, Mean ± SD	Min, Max	Median, Mean ± SD	Min, Max	Median, Mean ± SD	Min, Max
0.1	4, 4 ± 1	1, 5	3, 3 ± 1	2, 5	5, 4 ± 2	0.1, 6	2, 2 ± 0.6	0.9, 3
0.2	7, 7 ± 3	0.3, 11	4, 4 ± 2	1, 8	8, 7 ± 3	0.3, 11	3, 3.2 ± 2	0.6, 6
0.3	10, 9 ± 4	0.3, 15	7, 7 ± 3	3, 11	11, 10 ± 5	0.3, 18	5, 6 ± 2	2, 10
0.4	11, 9 ± 4	0.7, 15	9, 7 ± 3	2, 11	12, 10 ± 5	0.6, 16	7, 7 ± 2	2, 10
0.5	12, 11 ± 5	0.2, 18	9, 9 ± 3	1, 12	13, 12 ± 6	0.3, 22	8, 8 ± 3	1, 13

1	15, 14 ± 5	2, 23	10, 10 ± 5	2, 19	17, 14 ± 7	0.6, 25	10, 11 ± 5	3, 18
2	19, 18 ± 7	3, 26	12, 12 ± 6	2, 24	21, 18 ± 8	2, 29	13, 13 ± 7	2, 25
3	18, 20 ± 4	13, 27	18, 19 ± 7	8, 29	21, 18 ± 7	2, 28	16, 17 ± 8	4, 30
4	23, 23 ± 4	17, 28	21, 20 ± 6	12, 30	23, 22 ± 6	2, 29	20, 20 ± 7	10, 30
5	24, 24 ± 3	17, 30	29, 24 ± 9	12, 33	23, 22 ± 5	11, 31	23, 22 ± 9	11, 32

Supplementary Note 4

We have evaluated n_S by considering the effect of C_{IT}. After evaluating C_{IT} from the subthreshold slope, Supplementary Equation (S4) was used to evaluate n_S in the ON-state.

$$n_\mathrm{S} = \frac{C_\mathrm{G}}{q}\left(V_\mathrm{GS} - V_{t_\mathrm{lin}}\right); \quad C_\mathrm{G} = \frac{C_\mathrm{OX}\,(C_\mathrm{IT} + C_\mathrm{S})}{C_\mathrm{OX} + C_\mathrm{IT} + C_\mathrm{S}}; \quad C_\mathrm{S} = \frac{q^2\, n_\mathrm{S}}{k_\mathrm{B} T} \qquad (S4)$$

Here, C_G is the total gate capacitance. n_S = 10^{13} cm^{-2} for MoS_2 and n_S = 4.4 × 10^{12} cm^{-2} for WS_2 used in the main text corresponds to C_S of 6.13 × 10^{-1} $F.m^{-2}$ and 2.7 × 10^{-1} $F.m^{-2}$ respectively. Maximum C_IT is found to be 2.5 × 10^{-2} $F.m^{-2}$ and 1.7 × 10^{-2} $F.m^{-2}$ for MoS_2 and WS_2, respectively and C_OX = 1.6 × 10^{-3} $F.m^{-2}$. Hence, in the ON-state $C_\mathrm{S} \gg C_\mathrm{IT}$ as well as $C_\mathrm{S} \gg C_\mathrm{OX}$, resulting in $C_\mathrm{G} \approx C_\mathrm{OX}$, simplifying Supplementary Equation (S4) into Supplementary Equation (S5).

$$n_\mathrm{S} = \frac{C_\mathrm{OX}}{q}\left(V_\mathrm{GS} - V_{\mathrm{t_{lin}}}\right) \qquad (S5)$$

To obtain a constant n_S, a constant overdrive voltage $\left(V_\mathrm{GS} - V_{\mathrm{t_{lin}}}\right)$ is ensured by extracting the $V_{\mathrm{t_{lin}}}$ and then estimating the required V_GS for every device. I_ON is extracted from the output characteristics with a V_GS step size of 2 V, and the median error in n_S is 0.11×10^{12} cm^{-2} and 0.03×10^{12} cm^{-2} for MoS_2 and WS_2 FETs, respectively. n_S is also found for the analysis of R_c. R_c is extracted from the transfer characteristics with a V_GS step size of 85 mV, and median error in n_S is 0.003 and 0.004×10^{12} cm^{-2} for MoS_2 and WS_2 FETs, respectively.

Supplementary Table 6

Device-to-device variation in TLM mobility				
	MoS_2		WS_2	
	Median, Mean ± SD	Min, Max	Median, Mean ± SD	Min, Max
$\mu_{\mathrm{TLM}}(cm^2\ V^{-1}s^{-1})$	27.3, 27.7 ± 5.5	21.1, 46.5	16.2, 17.9 ± 9.7	2, 33.3

Supplementary Figure 6

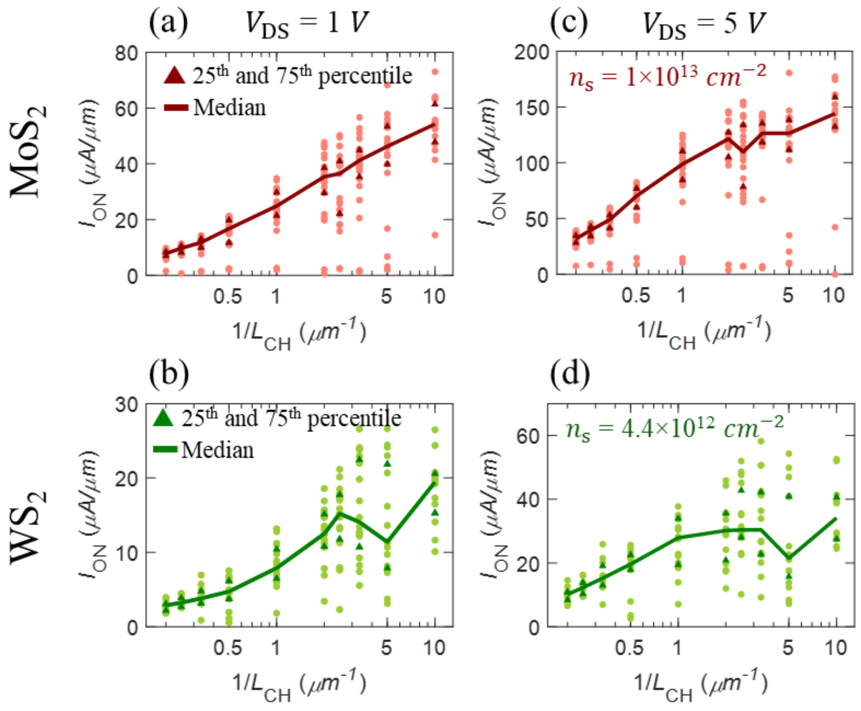

Supplementary Figure 6. Channel length dependence of drive current. Distribution of I_{ON} as a function of channel length (L_{CH}) for a) MoS_2 and b) WS_2 FETs for V_{DS} of 1 V, and for c) MoS_2 and d) WS_2 FETs for V_{DS} of 5 V. Note that the distribution is plotted as a function of $1/L_{\mathrm{CH}}$. Median, 25th percentile and 75th percentile is denoted.

Supplementary Table 7

Device-to-device variation in the drive current								
$I_{ON}(\mu A.\mu m^{-1})$ at $V_{DS} = 1\ V$					$I_{ON}(\mu A.\mu m^{-1})$ at $V_{DS} = 5\ V$			
	MoS_2		WS_2		MoS_2		WS_2	
L_{CH} (μm)	Median, Mean ± SD	Min, Max	Median, Mean ± SD	Min, Max	Median, Mean ± SD	Min, Max	Median, Mean ± SD	Min, Max
0.1	54, 52 ± 13	14, 73	17, 18 ± 5	10, 26	146, 141 ± 32	42, 177	30, 34 ± 10	25, 53
0.2	46, 41 ± 18	2, 68	11, 13 ± 8	3, 27	126, 109 ± 47	9, 180	20, 25 ± 14	7, 50
0.3	41, 38 ± 14	1, 57	14, 15 ± 6	6, 24	126, 116 ± 38	6, 144	28, 30 ± 13	9, 51
0.4	36, 31 ± 14	2, 50	15, 14 ± 5	2, 20	110, 104 ± 41	7, 155	30, 31 ± 9	10, 49
0.5	35, 32 ± 12	1, 48	12, 12 ± 4	3, 19	121, 110 ± 37	4, 146	30, 28 ± 10	12, 46
1	25, 24 ± 8	2, 35	8, 8 ± 3	2, 13	99, 92 ± 29	11, 125	26, 25 ± 8	7, 35
2	17, 16 ± 5	2, 21	5, 4 ± 2	1, 8	70, 64 ± 20	9, 82	20, 18 ± 7	3, 26
3	12, 11 ± 4	1, 14	4, 4 ± 1	1, 6	49, 45 ± 15	5, 60	15, 15 ± 4	7, 20
4	10, 9 ± 2	1, 11	3, 3 ± 1	2, 4	40, 37 ± 8	8, 46	12, 12 ± 3	6, 16
5	8, 7 ± 2	2, 10	3, 3 ± 1	1, 4	32, 31 ± 7	8, 39	10, 10 ± 3	5, 15

Supplementary Table 8

Benchmarking of scaling impact on MoS_2 FETs (parentheses show the number of devices used for the study)					
	$\mu_{g_m} (cm^2\ V^{-1} s^{-1})$			$I_{ON} (\mu A. \mu m^{-1})$	
	Our work	[3]	[4]	Our work	[3]
L_{CH} (μm)	Median, Mean ± SD	Median, Mean ± SD	Median, Mean ± SD	Median, Mean ± SD	Median, Mean ± SD
0.1	3.6, 4 ± 1 (17)	3, 2.7 ± 0.9 (17)		100, 99 ± 23 (17)	39.6, 36.7 ± 14.2 (17)
0.2	7.2, 6.6 ± 3.1 (22)	5, 4.6 ± 1.7 (24)		87, 78± 33 (22)	28.4, 27.1 ± 12.2 (24)
0.3	9.5, 8.9 ± 3.7 (22)			79, 72 ± 25 (23)	
0.4	11.3, 9.1 ± 4.3 (23)			69, 59 ± 26 (23)	
0.5	11.7, 10.8 ± 5.1 (23)	9.2, 8.5 ± 3 (27)		66, 62 ± 22 (23)	25.1, 22.3 ± 10 (27)
1	14.7, 14.2 ± 5.4 (21)	11.1, 10.5 ± 3.4 (27)		49, 46 ± 15 (23)	12.1, 12.9 ± 5 (27)
2	19.3, 17.8 ± 7.3 (21)			32, 30 ± 10 (22)	
3	17.8, 20.1 ± 4 (15)			23, 21 ± 7 (19)	
4	22.5, 23.1 ± 3.6 (19)		Nil, 34.2 ± 3.6 (200) L_{CH} = 4-8.6 μm	19, 17 ± 4 (20)	
5	23.9, 24.2 ± 3.1 (17)			15, 14 ± 3 (19)	

Supplementary Figure 7

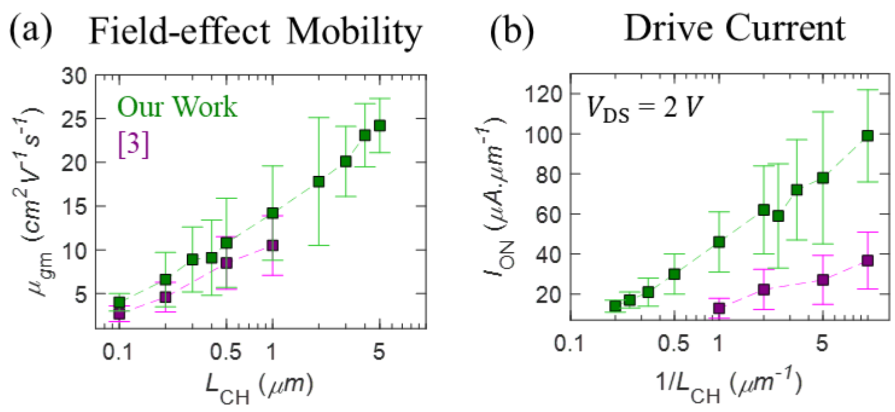

Supplementary Figure 7. Benchmarking scaling of MoS_2 FETs. Channel length dependent statistics with literature for a) field-effect mobility and b) drive current for synthetic monolayer MoS_2 FETs. Using error bar plots, mean and standard deviation is shown.

Supplementary Table 9

Device-to-device variation in saturation velocity				
	MoS_2		WS_2	
	Median, Mean ± SD	Min, Max	Median, Mean ± SD	Min, Max
v_{SAT} $(cm.sec^{-1})$ (10^5)	6.4, 5.9 ± 2.5	0.2, 11.2	4, 4.1 ± 1.5	1, 6.9

Supplementary References

[1] Q. Smets, B. Groven, M. Caymax, I. Radu, G. Arutchelvan, J. Jussot, *et al.*, "Ultra-scaled MOCVD MoS2 MOSFETs with 42nm contact pitch and 250µA/µm drain current," pp. 23.2.1-23.2.4, 2019.

[2] C. Min, T. Kamins, P. V. Voorde, C. Diaz, and W. Greene, "0.18-µm fully-depleted silicon-on-insulator MOSFET's," *IEEE Electron Device Letters,* vol. 18, pp. 251-253, 1997.

[3] H. Liu, M. Si, S. Najmaei, A. T. Neal, Y. Du, P. M. Ajayan, *et al.*, "Statistical study of deep submicron dual-gated field-effect transistors on monolayer chemical vapor deposition molybdenum disulfide films," *Nano Lett,* vol. 13, pp. 2640-6, Jun 12 2013.

[4] K. K. H. Smithe, S. V. Suryavanshi, M. Munoz Rojo, A. D. Tedjarati, and E. Pop, "Low Variability in Synthetic Monolayer MoS2 Devices," *ACS Nano,* vol. 11, pp. 8456-8463, Aug 22 2017.
